# The Effect of Strain Hardening on the Dynamic Response of Human Artery Segments

**DOI:** 10.2174/1874120701711010085

**Published:** 2017-09-26

**Authors:** Haralambia P. Charalambous, Panayiotis C. Roussis, Antonios E. Giannakopoulos

**Affiliations:** 1 Department of Civil & Environmental Engineering, University of Cyprus, Nicosia, Cyprus; 2 Department of Civil Engineering, University of Thessaly, Volos, Greece

**Keywords:** Hyperelastic arterial model, Human artery segments, Strain hardening, Dynamic analysis, Energy density

## Abstract

**Background:**

When subjected to time-dependent blood pressure, human arteries undergo large deformations, exhibiting mainly nonlinear hyperelastic type of response. The mechanical response of arteries depends on the health of tissues that comprise the artery walls. Typically, healthy arteries exhibit convex strain hardening under tensile loads, atherosclerotic parts exhibit stiffer response, and aneurysmatic parts exhibit softening response. In reality, arterial dynamics is the dynamics of a propagating pulse, originating in heart ventricle, propagating along aorta, bifurcating, *etc*. Artery as a whole cannot be simulated as a lump ring, however its cross section can be simulated as a vibrating ring having a phase lag with respect to the other sections, creating a running pressure wave. A full mathematical model would require fluid-solid interaction modeling continuity of blood flow in a compliant vessel and a momentum equation. On the other hand, laboratory testing often uses small-length arteries, the response of which is covered by the present work. In this way, material properties that change along the artery length can be investigated.

**Objective:**

The effect of strain hardening on the local dynamic response of human arteries (excluding the full fluid-structure interaction) is examined through appropriate hyperelastic models related to the health condition of the blood vessel. Furthermore, this work aims at constituting a basis for further investigation of the dynamic response of arteries accounting for viscosity.

**Method:**

The governing equation of motion is formulated for three different hyperelastic material behaviors, based on the constitutive law proposed by Skalak *et al.*, Hariton, and Mooney-Rivlin, associated with the hardening behavior of healthy, atherosclerotic, and aneurysmatic arteries, respectively. The differences between these modelling implementations are caused by physiology, since aneurysmatic arteries are softer and often sclerotic arteries are stiffer than healthy arteries. The response is investigated by proper normalization of the involved material parameters of the arterial walls, geometry of the arteries, load histories, time effects, and pre-stressing. The effect of each problem parameter on the arterial response has been studied. The peak response of the artery segment is calculated in terms of radial displacements, principal elongations, principal stresses, and strain-energy density. The validity of the proposed analytical models is demonstrated through comparison with previous studies that investigate the dynamic response of arterial models.

**Results:**

Important metrics that can be useful to vascular surgery are the radial deformation and the maximum strain-energy density along with the radial resonance frequencies. These metrics are found to be influenced heavily by the nonlinear strain-hardening characteristics of the model and the longitudinal pre-stressing.

**Conclusion:**

The proposed formulation permits a systematic and generalizable investigation, which, together with the low computational cost of analysis, makes it a valuable tool for calculating the response of healthy, atherosclerotic, and aneurysmatic arteries. The radial resonance frequencies can explain certain murmures developed in stenotic arteries.

## 1 INTRODUCTION

A simple way to view the human circulatory system is to think of a pump (heart) and a number of conduits (artery, veins) that circulate the blood [1]. The heart is basically a muscle that (normally) contrasts cyclically, resulting in pulsatile blood flow. Accordingly, the blood pressure rises to a peak (heart contrasts) and then falls (heart refills) during the cardiac circle. As blood pressure moves away from the heart, the systolic peak decreases and the diastolic value increases. The vascular elasticity is an important aspect of blood-flow dynamics. Arteries exhibit nonlinear mechanical behavior when extended, with their stiffness depending on strain [2]. Typically, their stiffness is monotonically increasing with strain, thereby protecting the artery from aneurysms and other instabilities under increasing pressure. Arteries are composite structures formed of tissues made of soft rubber-like proteins (elastin) reinforced by stiff collagen fibers. At a meso-level, arteries are formed by inhomogeneous layers, exhibit anisotropy, and have residual stresses. Furthermore, arteries show a viscoelastic response which accounts for a relatively low-energy loss in each inflation-deflation cycle, preventing reflected pressure waves from resonating in the arterial systems [3].

The stress-strain relationship of an arterial tissue exhibits anisotropic nonlinear behavior for finite deformations [4, 5]. The arterial tissue can be modeled as a hyperelastic material and as such its stress-strain relationship derives from a strain-energy function. Some, relatively simple, representative constitutive laws that describe the mechanical behavior of biological tissues are the Mooney-Rivlin [6, 7], Fung [8], Gent [9], the strain-energy function of Skalak *et al*. [10], and the constitutive law proposed by Delfino *et al*. [11]. Even though more sophisticated constitutive laws have been developed in recent years, they require a multitude of parameters that are not easy to be measured and are harder to be parametrized (see for example the model proposed by Holzapfel *et al*. [12]). Investigation of straight short artery segments requires dynamic cyclic inflation testing and models that use hyperelastic constitutive laws that are able to reproduce the test results.

In fact, there are several references in the literature suggesting that the use of too complex hyperelastic laws may be very difficult in revealing the generality of results and underlining the most important aspects of the problem. Humphrey and Na [13] observed that the more complex the arterial model, the less complex the stress field appears to be. Moreover, Hariton [14] modeled the realistic orientation of collagen fibers of the arterial tissue and observed that there is no significant difference regarding the macroscopic response of a simplified model that does not include the fiber orientation anisotropy.

To the authors’ best knowledge, very few publications are available in the literature that address analytically the dynamic response of arteries. The most representative studies are perhaps the works of Demiray and Vito [15] and Humphrey and Na [13] which both investigated the case of an exponential hyperelastic constitutive law. However, they assumed the spatial dependence of the deformation field in order to solve the problem.

Several quantitative methods for arterial wall function have been suggested, with the most popular ones being the pulse wave velocity and the flow-mediated vasodilatation [16]. Regarding the pulse wave velocity methodology, several one-dimensional models have been suggested to assess the propagation of the pressure pulse along the artery (see for example Pedley [17] and Formaggia *et al*. [18], for recent advancements of the mathematical modeling). It should be noted, however, that the aforementioned models presuppose the solution of the radial deformation of the arteries with respect to time. Taylor and Humphrey [19] pointed out that among the open problems in computational vascular biomechanics is the development of more robust techniques to create analytic models in order to include the general characteristic bevariors and provide predictive capability for artery failures.

Some investigations suggest that inertial effects appear insignificant for forcing frequencies less than 10 Hz (see for example David and Humphrey [20]). However, this is not at all the general case. Foreman and Hutchison [21] measured natural resonant vibration characteristics of the artery walls with stenosis. Stenosis excites the artery wall to vibrate over a wide range of frequencies within which are discrete resonant frequencies, the highest of which was recorded to be 550 Hz.

The aim of this study is to investigate the effect of strain hardening on the dynamic deformation of the artery cross-section, assuming three different physical behaviors: (a) hardening behavior of healthy arteries, (b) hardening behavior of atherosclerotic arteries, and (c) softening behavior of aneurysmatic arteries. These factors are investigated by adopting the following hyperelastic laws for each case respectively: (a) the constitutive law proposed by Skalak *et al*. [10], (b) the constitutive law of Delfino *et al*. [11], as modified by Hariton [14], and (c) the Mooney-Rivlin hyperelastic material [6, 7].

Fig. (**1**) plots the stress-strain relationships of the three nonlinear constitutive laws for typical values of their material parameters, in the absence of longitudinal pre-stretch $\left(\lambda_{z}^{0}=1\right)$ .

Note that the artery segments are assumed to be in taut-state of stress and under pre-stressed condition. The longitudinal (along the blood flow) pre-stress is accounted for explicitly, whereas other pre-stress effects are assumed to be incorporated in the constitutive law. An important side result of this work is the effect of loading and prestretch to the radial resonance frequencies of the aforementioned models. Radial resonance can explain murmures (acoustic sounds) developed in stenotic arteries followed by atheromatic plaque deterioration. Radial dynamic effects (impulse pressure) were found to be dangerous to aneurysmatic arteries because of the high deformations and stresses that may develop due to strain softening and thinning of the artery walls.

## 2 METHODS

The mathematical formulation is based on the following assumptions: (a) vessel cross-section in the undeformed state forms a full circle with thickness-averaged radius R; (b) the arterial wall has constant thickness along the circle and is small compared to the internal radius of the vessel; (c) the ring deforms radially only, under plain strain, incompressible conditions; (d) the arterial tissue consists of a single homogeneous layer; and (e) viscous effects are ignored.

Herein, the radius, thickness, and length of the initial configuration are denoted by *R, H, L* respectively; and the radius, thickness, and length of the deformed configuration are denoted by *r, h, l* respectively. Fig. (**2a**) shows the configuration of the idealized arterial model at the deformed state.

By considering the force equilibrium along the radial direction of the infinitesimal element *abcd* shown in Fig. (**2b**), the equation of motion of the deformed configuration is obtained as a balance of forces (including inertia):

(1) $$r\left(t\right)p\left(t\right)-N\left(t\right)=\rho \left(t\right)r\left(t\right)\frac{d^{2}u_{r}\left(t\right)}{dt^{2}}$$

where *ρ* denotes the density of the arterial tissue, *p*(*t*) is the uniform internal pressure, *N*(*t*) is the circumferential force that can be derived from a suitable hyperelastic constitutive law, and *u_r_*(*t*) is the radial displacement. Note that, due to the mass conservation and incompressibility of the arterial tissue, the initial density of the artery *ρ_0 _* is equal to the density of the artery at the deformed state *ρ* (*ρ = ρ_0 _*). The deformed radius can be expressed as:

(2) $$r\left(t\right)=R+u_{r}\left(t\right)$$

For incompressible materials (such as the arterial walls and many artificial grafts), the determinant of the deformation gradient is given by:

(3) $$\left|F\right|=J=\lambda_{\theta }\left(t\right)\lambda_{r}\left(t\right)\lambda_{z}^{0}=1$$

where *λ_θ _*(*t*) = *r*(*t*)/*R* is the elongation (stretch) of the circumferential direction, $\lambda_{z}^{0}=l/L$ is the elongation of the axial direction (considered to be the constant pre-stretch value caused by longitudinal residual stresses), and *λ_r_*(*t*) = *h(t)*/*H* is the elongation in the radial direction. Substituting in Eq. (3) we obtain,

(4) $$r\left(t\right)h\left(t\right)=\frac{\mathrm{RH}}{\lambda_{z}^{0}}$$

The artery is subjected to uniform intraluminal pressure. Fig. (**3a**) plots the aortic pressure-time profile as proposed by Zhong *et al*. [22, 16]. A normal cardiac cycle consists of a systolic (0 ≤ *t* ≤ *t*_s_) and a diastolic phase (*t_s_ < t ≤ t_cp_*). The aortic pressure-time profile is approximated in this study by the loading shown in Fig. (**3b**), representing the most conservative loading scenario [23, 17]. The value of the maximum systolic pressure is *p_s_* = 120 mmHg = 16 kPa, the diastolic pressure is *p_d_* = 80mmHg = 10.66kPa, the systolic-phase duration is *t_s_* = 0.35 sec, and the total duration of the cardiac pulse is *t_cp_* = 1 sec.

The following sections present the mathematical formulation of the arterial models for the three constitutive laws adopted in this study.

### 2.1 Arterial Model Based on the Strain-Energy Function of Skalak *et al*. (Hardening Behavior of Healthy Arteries)

To investigate the response of a healthy artery, we adopt the two-dimensional strain-energy function of Skalak *et al*. [10]. This strain-energy function, originally developed for red blood cell membranes, demonstrates hardening behavior. The strain-energy function proposed by Skalak *et al*. is given by:

(5) $$W\left(t\right)=\frac{B}{4}\left(\frac{1}{2}{\left(I\left(t\right)\right)}^{2}+I\left(t\right)-\mathrm{II}\left(t\right)\right)+\frac{C}{8}{\left(\mathrm{II}\left(t\right)\right)}^{2}$$

where *B* and *C* are the material properties of the artery, having units of elastic modulus multiplied by artery thickness [N/m], satisfying the condition *C ≥ B ≥ 0 and I(*t*) and II(*t*)* are alternative forms of the strain invariants, expressed as:

(6) $$I\left(t\right)=2\left(e_{\theta \theta }\left(t\right)+e_{\mathrm{zz}}\right)={\left(\lambda_{\theta }\left(t\right)\right)}^{2}+{\left(\lambda_{z}^{0}\right)}^{2}-2$$

(7) $$\mathrm{II}\left(t\right)=4I_{1}\left(t\right)=4e_{\theta \theta }\left(t\right)e_{\mathrm{zz}}+2\left(e_{\theta \theta }\left(t\right)+e_{\mathrm{zz}}\right)={\left(\lambda_{\theta }\left(t\right)\right)}^{2}{\left(\lambda_{z}^{0}\right)}^{2}-1$$

in which *e_θθ_* and *e_zz_* are the Green strain tensors.

The circumferential and longitudinal Cauchy stresses, multiplied by the current artery thickness, are obtained respectively as:

(8) $$\sigma_{\theta \theta }\left(t\right)h\left(t\right)=T_{\theta }\left(t\right)=\frac{\lambda_{\theta }\left(t\right)}{\lambda_{z}^{0}}\frac{\partial W}{\partial e_{\theta \theta }}=\frac{\lambda_{\theta }\left(t\right)}{\lambda_{z}^{0}}\left[\frac{B}{2}\left({\left(\lambda_{\theta }\left(t\right)\right)}^{2}-1\right)+\frac{C}{2}{\left(\lambda_{z}^{0}\right)}^{2}\left({{\left(\lambda_{\theta }\left(t\right)\right)}^{2}\left(\lambda_{z}^{0}\right)}^{2}-1\right)\right]$$

(9) $$\sigma_{\mathrm{zz}}\left(t\right)h\left(t\right)=T_{z}=\frac{\lambda_{z}^{0}}{\lambda_{\theta }\left(t\right)}\frac{\partial W}{\partial e_{\mathrm{zz}}}=\frac{\lambda_{z}^{0}}{\lambda_{\theta }\left(t\right)}\left[\frac{B}{2}\left({\left(\lambda_{z}^{0}\right)}^{2}-1\right)+\frac{C}{2}{\left(\lambda_{\theta }\left(t\right)\right)}^{2}\left({{\left(\lambda_{\theta }\left(t\right)\right)}^{2}\left(\lambda_{z}^{0}\right)}^{2}-1\right)\right]$$

Note that, for *B/C = 0* the circumferential and longitudinal normalized stresses are equal.

The axial force acting along the circumferential direction *N*(*t*) is identical to *T_θ_* (*t*) (*N* (*t*) = *T* (*t*)). Therefore, by substituting Eqs. (2), (4), and (8) in Eq. (1), the normalized equation of motion governing the arterial response becomes a non-linear differential equation for the radial displacement *u_r_* (*t*):

(10) $$-\frac{u_{r}\left(t\right)}{R}\left[\frac{B}{C\lambda_{z}^{0}}+\frac{3{\left(\lambda_{z}^{0}\right)}^{3}}{2}-\frac{\lambda_{z}^{0}}{2}\right]-{\left(\frac{u_{r}\left(t\right)}{R}\right)}^{2}\left[\frac{3B}{2C\lambda_{z}^{0}}+\frac{{3\left(\lambda_{z}^{0}\right)}^{3}}{2}\right]-{\left(\frac{u_{r}\left(t\right)}{R}\right)}^{3}\left[\frac{B}{2C\lambda_{z}^{0}}+\frac{{\left(\lambda_{z}^{0}\right)}^{3}}{2}\right]+\left[\left(1+\frac{u_{r}\left(t\right)}{R}\right)p\left(t\right)\frac{R}{C}-\frac{{\left(\lambda_{z}^{0}\right)}^{3}}{2}+\frac{\lambda_{z}^{0}}{2}\right]=\frac{\rho_{0}R^{2}H}{C\lambda_{z}^{0}}\frac{{\overset{..}{u}}_{r}\left(t\right)}{R}$$

Observe that the mathematical formulation of the healthy artery involves six dimensionless quantities: B/C, λz0, ptR/C, urt/R, tSku..r2t/R, and t/tSk. The term tSk=ρ0R2H/Cλz0 represents the characteristic time of the response.

Equation (10) can be solved for four complexity levels, each one having a different order of nonlinearity (zero-, first-, second-, and third-order). The “third-order nonlinear” equation stands for the fully nonlinear problem described in Eq. (10). The “zero-order nonlinear” equation is a second-order linear non-homogeneous differential equation with constant coefficients. To obtain the “zero-order nonlinear” equation, we neglect the second- and third-power terms of radial displacement and the term *p*(*t*)u_r_ (*t*) / *R* of Eq. (10), resulting in

(11) $$\left[p\left(t\right)\frac{R}{C}-\frac{{\left(\lambda_{z}^{0}\right)}^{3}}{2}+\frac{\lambda_{z}^{0}}{2}\right]-\frac{u_{r}\left(t\right)}{R}\left[\frac{B}{C\lambda_{z}^{0}}+\frac{3{\left(\lambda_{z}^{0}\right)}^{3}}{2}-\frac{\lambda_{z}^{0}}{2}\right]=\frac{\rho_{0}R^{2}H}{C\lambda_{z}^{0}}\frac{{\overset{..}{u}}_{r}\left(t\right)}{R}$$

For $\lambda_{z}^{0}=1$, Eq. (11) is identical to the equation of motion of the linear model. The natural circular frequency (for *p*(*t*) = 0) of the “zero-order nonlinear” equation is given by:

(12) $$\omega_{0}^{\mathrm{Sk}}=\frac{\sqrt{\frac{B}{C\lambda_{z}^{0}}+\frac{3}{2}{\left(\lambda_{z}^{0}\right)}^{3}-\frac{\lambda_{z}^{0}}{2}}}{t_{\mathrm{Sk}}}$$

Equation (12) implies that periodic solutions $\left(\omega_{0}^{\mathrm{sK}}\;\mathrm{Real}\right)$ are possible if $\lambda_{z}^{0}\geq 1$ (which is physically true for most healthy arteries) or *C ≤ *24* B*. If these inequalities are not observed, then it is possible to have material instability. We are particularly interested in the artery response in terms of the circumferential elongation λ_θ_, current thickness *h*, circumferential stress *T_θ_*, longitudinal stress *T_z_*, and strain-energy density values *W*. The normalized functions for these response quantities can be synopsized as:

(13) $$\lambda_{\theta }\left(t\right)=1+\frac{u_{r}\left(t\right)}{R}$$

(14) $$\frac{h\left(t\right)}{H}=\frac{1}{\lambda_{\theta }\left(t\right)\lambda_{z}^{0}}$$

(15) $$\frac{T_{\theta }\left(t\right)}{C}=\frac{\lambda_{\theta }\left(t\right)}{\lambda_{z}^{0}}\left[\frac{B}{2C}\left({\left(\lambda_{\theta }\left(t\right)\right)}^{2}-1\right)+\frac{{\left(\lambda_{z}^{0}\right)}^{2}}{2}\left({{\left(\lambda_{\theta }\left(t\right)\right)}^{2}\left(\lambda_{z}^{0}\right)}^{2}-1\right)\right]$$

(16) $$\frac{T_{z}\left(t\right)}{C}=\frac{\lambda_{z}^{0}}{\lambda_{\theta }\left(t\right)}\left[\frac{B}{2C}\left({\left(\lambda_{z}^{0}\right)}^{2}-1\right)+\frac{{\left(\lambda_{\theta }\left(t\right)\right)}^{2}}{2}\left({\left(\lambda_{\theta }\left(t\right)\right)}^{2}{\left(\lambda_{z}^{0}\right)}^{2}-1\right)\right]$$

(17) $$\frac{W\left(t\right)}{C}=\frac{B}{4C}\left(\frac{1}{2}{\left(I\left(t\right)\right)}^{2}+I\left(t\right)-\mathrm{II}\left(t\right)\right)+\frac{{\left(\mathrm{II}\left(t\right)\right)}^{2}}{8}$$

Note that, Eq. (14) is obtained by solving Eq. (3) for *h* (*t*)/*H*, and Eqs. (15) through (17) are obtained by normalizing Eqs. (8), (9), and (5), respectively, by the (non-zero) material parameter *C*.

### 2.2 Arterial Model Based on the Strain-Energy Function of Hariton (Hardening Behavior of Atherosclerotic Arteries)

Atherosclerotic arteries are stiffer than healthy arteries. To study the response of an atherosclerotic artery that demonstrates exponential hardening, we adopt the isotropic, three-dimensional strain-energy function proposed by Hariton [14], which is a modification of the strain-energy function proposed by Delfino *et al*. [11]. The strain-energy function proposed by Hariton is given by

(18) $$W\left(t\right)=\frac{a}{b}\left\{\exp\left[\frac{b}{2}{\left(I_{1}\left(t\right)-3\right)}^{2}\right]-1\right\}$$

where *a* > 0 is a stress-like parameter, and *b* > 0 is a non-dimensional material parameter. Typical values of the material parameters are *a* = 44.2 kPa and *b* = 16.7 [11]. Additionally, $I_{1}\left(t\right)={\left(\lambda_{\theta }\left(t\right)\right)}^{2}+{\left(\lambda_{z}^{0}\right)}^{2}+1/{\left(\lambda_{z}^{0}\lambda_{\theta }\left(t\right)\right)}^{2}$ is the first strain invariant.

The Cauchy stress-strain relationships of the circumferential and longitudinal directions are then obtained as

(19) $$\sigma_{\theta \theta }\left(t\right)=\lambda_{\theta }\left(t\right)\frac{\partial W}{\partial \lambda_{\theta }\left(t\right)}-P\approx 2a\left[{\left(\lambda_{\theta }\left(t\right)\right)}^{2}-\frac{1}{{\left(\lambda_{\theta }\left(t\right)\lambda_{z}^{0}\right)}^{2}}\right]\left(I_{1}\left(t\right)-3\right)\exp\left[\frac{b}{2}{\left(I_{1}\left(t\right)-3\right)}^{2}\right]$$

(20) $$\sigma_{\mathrm{zz}}\left(t\right)=\lambda_{z}^{0}\left(t\right)\frac{\partial W}{\partial \lambda_{z}^{0}}-P\approx 2a\left[{\left(\lambda_{z}^{0}\right)}^{2}-\frac{1}{{\left(\lambda_{\theta }\left(t\right)\lambda_{z}^{0}\right)}^{2}}\right]\left(I_{1}\left(t\right)-3\right)\exp\left[\frac{b}{2}{\left(I_{1}\left(t\right)-3\right)}^{2}\right]$$

where *P* is the hydrostatic pressure (due to incompressibility). For a thin-wall assumption and a stress-free outer surface, *P ≈ 0*.

The circumferential arterial force is equal to the circumferential stress multiplied by the current thickness $\left(N\left(t\right)\right)=\sigma_{\theta \theta }\left(t\right)h\left(t\right)$ On substituting Eqs. (2), (4), and (19) in Eq. (1) the normalized equation of motion governing the arterial response becomes

(21) $$\frac{\rho_{0}R^{2}}{a\lambda_{z}^{0}}\frac{{\overset{..}{u}}_{r}\left(t\right)}{R}=-\frac{2}{\lambda_{z}^{0}}\left[\left(1+\frac{u_{r}\left(t\right)}{R}\right)+\frac{1}{{\left(\lambda_{z}^{0}\right)}^{2}{\left(1+\frac{u_{r}\left(t\right)}{R}\right)}^{3}}\right]\;\left[{\left(1+\frac{u_{r}\left(t\right)}{R}\right)}^{2}+{\left(\lambda_{z}^{0}\right)}^{2}+\frac{1}{{{\left(\lambda_{z}^{0}\right)}^{2}\left(1+\frac{u_{r}\left(t\right)}{R}\right)}^{2}}-3\right]\times \exp\left\{\frac{b}{2}{\left[{\left(1+\frac{u_{r}\left(t\right)}{R}\right)}^{2}+{\left(\lambda_{z}^{0}\right)}^{2}+\frac{1}{{{\left(\lambda_{z}^{0}\right)}^{2}\left(1+\frac{u_{r}\left(t\right)}{R}\right)}^{2}}-3\right]}^{2}\right\}+\left(1+\frac{u_{r}\left(t\right)}{R}\right)p\left(t\right)\frac{R}{\mathrm{aH}}$$

The six dimensionless quantities of this model are b, λz0, ptR/aH, urt/R,tH u..r2t/R, and t/tH, where the termtH=ρ0R2/aλz0 is the characteristic time of the response. Note that the natural frequency of the “zero-order nonlinear” equation is zero. Equation (21) is again a non-linear differential equation that should be solved for the radial displacement *u_r_* (*t*) as a function of time.

### 2.3 Arterial Model Based on the Strain-Energy Function of Mooney-Rivlin (Softening Behavior of Aneurysmatic Arteries)

Healthy arteries exhibit hardening with increased strain, whereas aneurysmatic arteries exhibit softening. The softening behavior of aneurysmatic arteries can be described by the isotropic three-dimensional constitutive law of Mooney-Rivlin. The strain-energy function suitable for incompressible materials has the following form [7]:

(22) $$W\left(t\right)=\frac{1}{2}\mu \left[\left(\frac{1}{2}+\beta \right)\left(I_{1}\left(t\right)-3\right)+\left(\frac{1}{2}-\beta \right)\left(I_{2}\left(t\right)-3\right)\right],\;\mu >0,\;-\frac{1}{2}\leq \beta \leq \frac{1}{2}$$

where $I_{2}\left(t\right)={\left(\lambda_{\theta }\left(t\right)\right)}^{2}{\left(\lambda_{z}^{0}\right)}^{2}+1/{{\left(\lambda_{z}^{0}\right)}^{2}+1/\left(\lambda_{\theta }\left(t\right)\right)}^{2}$ is the second strain invariant for incompressible materials, *µ* is the shear modulus of the material under infinitesimal deformation of the initial undeformed configuration, and *β* is a dimensionless material constant. The value *β* = 1/2 corresponds to the Neo-Hookean model.

The circumferential and longitudinal Cauchy stress-strain relationships can be obtained by using the equations of Chadwick [7] for the internal surface of the artery. Based on the thin-wall assumption, and in the absence of pressure in the outer wall, the radial stress is almost zero (σ_rr_ ≈ 0). Therefore, the stress-strain relations of the circumferential and longitudinal directions are approximated respectively by

(23) $$\sigma_{\theta \theta }\left(t\right)\approx \mu \left(\frac{1}{2}+\beta \right)\left({\left(\lambda_{\theta }\left(t\right)\right)}^{2}-\frac{1}{{\left(\lambda_{\theta }\left(t\right)\right)}^{2}{\left(\lambda_{z}^{0}\right)}^{2}}\right)+\mu \left(\frac{1}{2}-\beta \right)\left({\left(\lambda_{\theta }\left(t\right)\right)}^{2}{\left(\lambda_{z}^{0}\right)}^{2}-\frac{1}{{\left(\lambda_{\theta }\left(t\right)\right)}^{2}}\right)$$

(24) $$\sigma_{\mathrm{ZZ}}\left(t\right)\approx \mu \left(\frac{1}{2}+\beta \right)\left({\left(\lambda_{z}^{0}\right)}^{2}-\frac{1}{{\left(\lambda_{\theta }\left(t\right)\right)}^{2}{\left(\lambda_{z}^{0}\right)}^{2}}\right)+\mu \left(\frac{1}{2}-\beta \right)\left({\frac{1}{{\left(\lambda_{z}^{0}\right)}^{2}}-\left(\lambda_{\theta }\left(t\right)\right)}^{2}{\left(\lambda_{z}^{0}\right)}^{2}\right)$$

Note that, in the absence of longitudinal pre-stretch, the material parameter *β* has no effect on the stress-strain behavior.

The normalized equation of motion of the Mooney-Rivlin arterial model is then obtained as

(25) $$-\frac{1}{\lambda_{z}^{0}}\left\{\left(\frac{1}{2}+\beta \right)\left[\left(1+\frac{u_{r}\left(t\right)}{R}\right)-\frac{1}{{\left(\lambda_{z}^{0}\right)}^{2}{\left(1+\frac{u_{r}\left(t\right)}{R}\right)}^{3}}\right]+\left(\frac{1}{2}-\beta \right)\left[\left(1+\frac{u_{r}\left(t\right)}{R}\right){\left(\lambda_{z}^{0}\right)}^{2}-\frac{1}{{\left(1+\frac{u_{r}\left(t\right)}{R}\right)}^{3}}\right]\right\}+\left(1+\frac{u_{r}\left(t\right)}{R}\right)\frac{\mathrm{Rp}\left(t\right)}{\mu H}=\frac{\rho_{0}R^{2}}{\mu \lambda_{z}^{0}}\frac{{\overset{..}{u}}_{r}\left(t\right)}{R}$$

In this case, the six dimensionless quantities of the Mooney-Rivlin arterial model are β, λz0, ptR/μH, urt/R, tMRu ..r2t/R, t/tMR, where the term $t_{\mathrm{MR}}=\sqrt{\rho_{0}R^{2}/\mu \lambda_{z}^{0}}$ corresponds to the characteristic time of the response of the Mooney-Rivlin arterial model.

The natural frequency (for *p(t)* = 0) of the equivalent “zero-order nonlinear” model is given by

(26) $$\omega_{0}^{\mathrm{MR}}=\frac{1}{t_{\mathrm{MR}}}\sqrt{\frac{2}{\lambda_{z}^{0}}-\frac{2\beta }{\lambda_{z}^{0}}+\frac{3}{2{\left(\lambda_{z}^{0}\right)}^{3}}+\frac{3\beta }{{\left(\lambda_{z}^{0}\right)}^{3}}+\frac{\lambda_{z}^{0}}{2}-\beta \lambda_{z}^{0}}$$

This result (that applies to Mooney-Rivlin nonlinear behavior) agrees with the classic results of Knowles [24] for λ_Z_^0 ^ = 1 (no prestretch) ω_0 _^MR^ = 2/*t*_MR_. Note that Eq. (26) predicts a real natural frequency for all values of *λ*_z_^0 ^ and *β*. The natural frequency increases with the prestretch λ_Z_^ 0^ ≥ 1.

#### 2.3.1 Numerical Solution

The nonlinear dynamic equations that describe the physical problem can be characterized, from the numerical point of view, as “stiff”, hence their solution demands special methods. An ordinary differential equation (ode) is “stiff”, when there are computational efficiency issues (large computational time) and the numerical method must drastically reduce the time step to obtain satisfactory results of the solution. In our case, the efficiency issues are caused due to the large differences in the order of magnitude of the differential equation coefficients.

The formulated differential equations can be solved numerically through the appropriate ode solvers in MATLAB [25, 26]. The problem is solved by transforming the second-order differential equation into two first-order equations (state-space analysis). The ordinary differential equations of the arterial models based on the strain-energy function of Skalak *et al.* (Eqs. (10) and (11)), and on the strain-energy function of Mooney-Rivlin (Eq. (25)) are solved numerically by using the ode23s function in MATLAB, a one-step solver based on the modified Rosenbrock method [27, 28]. The ordinary differential equation of the arterial model based on the strain-energy function of Hariton (Eq. (29)) is solved numerically by using the ode23tb solver in MATLAB. The ode23tb solver uses an implicit Runge-Kutta method [29], which is suitable for very stiff problems.

Furthermore, the problem has been investigated by proper normalization of the involved material parameters and of the pressure time-profile.

## 3 RESULTS

Representative analysis results are presented in this section in terms of response spectra for the three models adopted in this study: healthy, atherosclerotic, and aneurysmatic arteries. The maximum radial displacement of the arterial model, as well as the response spectra of the circumferential elongation, variation of thickness, circumferential stress, longitudinal stress, and strain-energy density are investigated by varying the problem parameters.

In order to compare these models with the linear arterial model, the initial tangent Young's modulus *E_θ_* of the respective case is substituted in the linear equation of motion [17, 24]:

(27) $$\rho_{0}H{\overset{..}{u}}_{r}\left(t\right)=p\left(t\right)-\frac{E_{\theta }H}{R^{2}}u_{r}\left(t\right)$$

The expressions giving the tangential circumferential Young's modulus *E_θ_* of the Skalak *et al.*, the Hariton, and the Mooney-Rivlin constitutive laws are obtained, respectively, as

(28) $$\begin{array}{l} E_{\theta }={\left.\frac{d\sigma_{\theta \theta }}{d\lambda_{\theta }}\right|}_{\lambda_{\theta \to 1}}={\left.\frac{d}{d\lambda_{\theta }}\left(\frac{T_{\theta }}{h}\right)\right|}_{\lambda_{\theta \to 1}}={\left.\frac{d}{d\lambda_{\theta }}\left(\frac{\lambda_{\theta }\lambda_{z}^{0}T_{\theta }}{H}\right)\right|}_{\lambda_{\theta \to 1}}=\frac{B}{H}+2\frac{C}{H}{\left(\lambda_{z}^{0}\right)}^{4}-\frac{C}{H}{\left(\lambda_{z}^{0}\right)}^{2}\\ E_{\theta }={\left.\frac{d\sigma_{\theta \theta }}{d\lambda_{\theta }}\right|}_{\lambda_{\theta \to 1}}=4a\left\{{\left(\lambda_{z}^{0}\right)}^{2}-\frac{3}{{\left(\lambda_{z}^{0}\right)}^{2}}+\frac{2}{{\left(\lambda_{z}^{0}\right)}^{4}}+b{\left(1-\frac{1}{{\left(\lambda_{z}^{0}\right)}^{2}}\right)}^{2}{\left({\left(\lambda_{z}^{0}\right)}^{2}+\frac{1}{{\left(\lambda_{z}^{0}\right)}^{2}}-2\right)}^{2}\right\} \end{array}$$

(29) $$\exp\left[\frac{b}{2}{\left({\left(\lambda_{z}^{0}\right)}^{2}+\frac{1}{{\left(\lambda_{z}^{0}\right)}^{2}}-2\right)}^{2}\right]$$

(30) $$E_{\theta }={\left.\frac{d\sigma_{\theta \theta }}{d\lambda_{\theta }}\right|}_{\lambda_{\theta \to 1}}=2\mu \left[\left(\frac{1}{2}+\beta \right)\left(1+\frac{1}{{\left(\lambda_{z}^{0}\right)}^{2}}\right)+\left(\frac{1}{2}-\beta \right)\left(1+{\left(\lambda_{z}^{0}\right)}^{2}\right)\right]$$

The solution of Eq. (27), for the dynamic loading of Fig. (**3b**), can be expressed in closed form [24, 17]. The longitudinal pre-tension of the linear model is taken into account through the initial displacement *u_r_*(0) = *u_0 _*. We assume that the initial displacement is *u*_0 _ = *R* (λ_Z_^ 0^ - 1) and the initial velocity is ${\dot{u}}_{r}\left(0\right)=0$ . Note that λ_Z_^0 ^ ≥ 1 for all cases (and so *E_θ_> * 0 for all models).

### 3.1 Response of Healthy Arteries

The total response of the Skalak model is obtained by solving the fully (“third-order”) nonlinear model. A comparison between the linear model, the “zero-order nonlinear” model, and the “third-order nonlinear” model is presented through radial-displacement spectra in Figs. (**4**) and (**5**).

Fig. (**4**) suggests that an increase of the longitudinal pre-stretch λ_Z_^ 0^ or the ratio *B/C*, stiffens the artery and decreases the radial displacement. For pre-stretch values between 1.1 and 1.15 (typical values for healthy arteries), the absolute value of the radial deformation is minimized (for *p_s_R/ C =* 0.16). However, for λ_Z_^ 0^ > 1.12 we observe that a negative radial displacement spectrum, indicating a possible reverse flow in the artery. The resonant frequency increases from 2000 s^-1^ at no prestretch (λ_Z_^ 0^ = 1) to 3200 s^-1^ at λ_Z_^ 0^ = 1.3. On the other hand, Fig. (**5**) shows that an increase in the normalized systolic pressure *p_s_R/ C*, yields increased radial deformation response (Fig. **5a**), whereas the characteristic time *t_Sk_* seems not to affect the problem at low levels of normalized pressure *p_s_R/C* (Fig. **5b**). Note that, for λ_Z_^ 0^ = 1, the linear and the “zero-order nonlinear” models yield identical response. Zeroth-order resonant frequency $\omega_{0}^{\mathrm{Sk}}$ : (a) increases from 2000 s^-1^ to 3200 s^-1^ with increasing λ_Z_^ 0^ from 1 to 1.3 (*B/C = *0, *p_s_R/C = *0.16, *t_cp_/t_Sk_* = 2000), (b) increases slightly from 2000 s^-1^ to 2800 s^-1^ with increasing *B/C* from 0 to 1 *(λ_Z_^ 0^* = 1, *p_s_R/C *= 0.16, *t_cp_/t_Sk_* = 2000), and (c) increases from 2000 s^-1^ to 9000 s^-1^ with increasing t_cp_/t_Sk_ (*B/C = *0, *λ_Z_^ 0^ = *1,* p_s_R/C *= 0.16).

It is evident that the most important parameters influencing the problem are the pre-stretch value λ_Z_^ 0^, the ratio *B/C* and the normalized systolic pressure *p_s_R/C*. Accordingly, the spectra of the response quantities of Eqs. (13) through (17) are investigated by varying the values of the aforementioned parameters. Fig. (**6**) presents response spectra as a function of the ratio *B/C* for three different values of parameter *p_s_ R / C*. It can be observed that the maximum response of the system is decreased with increasing values of *B/C* or with decreasing values of the normalized systolic pressure *p_s_ R / C*. Moreover, the “zero-order nonlinear” model seems to be conservative compared to the “third-order nonlinear” model.

Table **1**. lists the peak value of the normalized strain energy *W / C* along with the time of its occurrence for different longitudinal pre-stretch values, for three values of the ratio *B / C* and for *p_s_R / C = *0.16.

### 3.2 Response of Atherosclerotic Arteries

Figs. (**7**) and (**8**) plot the peak normalized radial deformations of atherosclerotic arterial systems (arterial model based on the strain-energy function of Hariton) for different values of four basic non-dimensional parameters (*λ_z_^ 0^, b, p_s_R/aH, t_cp_ / t_H_*). As can be seen from these figures, the artery becomes stiffer exhibiting reduced radial displacement as the longitudinal pre-stretch (Fig. **7a**) or the material parameter *b* (Fig. **7b**) is increased. Fig. (**9**) presents response spectra for different values of the material parameter *b* and for three different values of the normalized systolic pressure *p_s_R/aH*. An increase of the material parameter *b* results in a decrease of the circumferential elongation and normalized strain energy, and an increase of the normalized circumferential and longitudinal stresses.

Table **2** lists the peak values of normalized strain energy *W / a*, for different cases of longitudinal pre-stretch, for three values of the material parameter *b*, and for *p_s_R / aH = *3.2. Table **3** lists the peak values of the normalized strain energy *W / a*, for different cases of longitudinal pre-stretch and for three values of the normalized systolic pressure *p_s_R / aH*. Reported in Tables **2** and **3** is also the time instant at which the peak value of the normalized strain energy occurs.

### 3.3 Response of Aneurysmatic Arteries

This section compares the radial displacement of the Mooney-Rivlin arterial model with that of the equivalent linear model. All models in the analysis assume the same initial tangent elasticity modulus *E_θ_*. Furthermore, peak values of the respective response quantities (Eq. (13), Eq. (14), and Eqs. (22) through (24) normalized by the material parameter *µ*) are presented for different values of the non-dimensional parameters.

Figs. (**10**) and (**11**) plot the peak radial displacement as a function of the longitudinal pre-stretch $\lambda_{z}^{0}$ , the material parameter *β*, the normalized systolic pressure *p_s_R / µH*, and the normalized characteristic time *t_cp_ / t_MR_* for different values of the problem parameters. Fig. (**12**) present response spectra as a function of the material parameter *β*, for three values of the parameter *p_s_R / µH* and for $\lambda_{z}^{0}$ = 1.1.

Tables **4** and **5** report the time instant at which the peak value of normalized strain energy occurs. Again, in all cases, the peak value occurs during the systolic phase.

### 3.4 Numerical Examples

To illustrate the applicability of the proposed analytical models, numerical examples are presented and compared against studies that investigate the dynamic radial response of arterial models. Such studies are the works of Demiray and Vito [15] and Humphrey and Na [13], which both considered the case of an exponential hyperelastic constitutive law.

Demiray and Vito [15] studied the radial deformations of arteries subjected to dynamic inner pressure. They assumed an isotropic, homogeneous, incompressible artery obeying the exponential strain-energy density function of Blatz *et al.* [30]. They presented a numerical example based on data corresponding to a dog's abdominal aorta. The aorta, having inner radius 3.12 mm, outer radius 3.80 mm, longitudinal pre-stretch equal to 1.53, is subjected to dynamic loading with systolic and diastolic pressures 9.892 kPa and 3.466 kPa, respectively. According to their calculations, the circumferential stress at the artery centerline, at the beginning of the systolic phase, was 395.7 kPa.

Utilizing the data from the example of Demiray and Vito, we calculated the arterial response for the hyperelastic functions adopted in this study. The material parameters of each case were selected to have about the same initial tangent modulus and adequate curve fitting compared to the circumferential stress-strain curve of the analysis of Demiray and Vito (Fig. **13a**). Table **6** lists the data used for each model and the corresponding response values. The Hariton model yields a peak circumferential stress equal to 446.69 kPa, approximating well the respective value calculated by Demiray and Vito (395.7 kPa). The linear model and the Skalak model yield lower values of circumferential stress, whereas the Mooney-Rivlin arterial model is not suitable for the data (large pre-stretch value) of this example. Note that, the peak strain-energy density values corresponding to the Skalak model and Hariton model are comparable, whereas the linear model yields larger strain-energy density values.

Humphrey and Na [13] studied the dynamic response of an artery and the corresponding wall stresses. They assumed that the artery is anisotropic, homogeneous, incompressible and obeys the exponential hyperelastic law of Chuong and Fung [31]. They presented a numerical example on the passive response of an artery subjected to two cardiac cycles with systolic and diastolic pressures of 105 mmHg and 91 mmHg, respectively. The artery has inner radius 1.39 mm, outer radius 1.99 mm, and longitudinal pre-stretch 1.832. The model also accounted for residual circumferential stresses by using the approximate “opened-up” stress-free configuration [31]. The peak circumferential and axial stresses of the inner surface were calculated by Humphrey and Na as 212.8 kPa and 177 kPa, respectively (in general the inner surface has lower stress values than the outer surface). The maximum radial displacement of the outer surface was computed to be 0.72 mm (mean strain 42%).

Adopting the data from the example of Humphrey and Na, we investigated the arterial response for the hyperelastic functions considered in this study. The material parameters of each case were selected to have about the same initial tangent modulus and adequate curve fitting compared to the circumferential stress-strain curve of the analysis of Humphrey and Na (Fig. **13b**). To account for the residual circumferential stresses, we assumed that a compressive pressure equal to 50 mmHg is applied to the arterial wall. By calculating the difference, the systolic and diastolic pressures are 55 mmHg and 41 mmHg, respectively. Table **7** lists the data used for the linear, Skalak, and Hariton arterial model and the corresponding response values. Note that the Mooney-Rivlin hyperelastic function is not suitable for the data (large pre-stretch value) of this example.

It should be noted that our calculations are based on the average-stress assumption, whereas the values reported in Humhprey and Na refer to the stresses of the inner surface. The calculated values of the arterial model of Skalak (strain 55% and circumferential stress 153.22 kPa) approach better the results of Humprey and Na. Additionally, we can observe that there is a variation of the peak values of strain energy for the different material constitutive laws.

Based on these results, we can say that the response of the numerical examples presented in the aforementioned studies can be adequately approximated by the arterial models proposed in the present study (presupposing that the circumferential stress-strain curves adopted in the present study have sufficient curve fitting over the stress-strain curves of the numerical examples).

## DISCUSSION

The effect of strain hardening in the dynamic response of human arteries has been examined through appropriate models related to the health condition of the blood vessel.

Regarding the Shalak model we observe the followings. In cases of hypertension, the systolic pressure *p_s_* can be as high as 200 mmHg, *i.e.* 5/3 of the normal systolic pressure (120 mmHg). What does this mean for the radial response of a healthy artery? According to Fig. (**5a**), in case of hypertension, it would reach a value of *p_s_R/C* = 0.27 and the normalized radial displacement would increase from 28% to 42%. In the case that the elasticity modulus $\left(E_{\theta }={\left(B+2C\left(\lambda_{z}^{0}\right)\right)}^{4}-C{\left(\lambda_{z}^{0}\right)}^{2}/H\right)$ decreases, the material parameter *C/H* of the artery decreases. Considering that the material parameter *C/H* has typical values between 0.1 and 1 MPa, the normalized systolic pressure *p_s_R/C* is potentially increased by a factor of 10 for soft arteries, yielding a radial displacement response over 100%, as shown in Fig. (**5a**). In most cases, the peak strain-energy value occurs during the systolic phase. As expected, the circumferential elongation decreases with increasing values of the longitudinal pre-stretch $\lambda_{z}^{0}$, while the normalized strain energy and normalized stresses exhibit an optimized minimum value for $\lambda_{z}^{0}$ between 1.1 and 1.15. When $\lambda_{z}^{0}$ is increased over this optimized value, the normalized strain energy is increased rapidly, indicating possible failure for pre-stretch values close to 1.3. For increasing values of the ratio *B/C*, the response decreases for pre-stretch values up to 1.1-1.15, and increases for higher pre-stretch values.

Regarding the Hariton model we observe the followings. The normalized radial displacement increases slightly with increasing values of the normalized systolic pressure *p_s_R/ aH* (Fig. **8a**), whereas the characteristic time *t_H_* seems not to affect the problem (Fig. **8b**). As expected, the linear model yields larger normalized radial displacements compared to the hyperelastic model, except for large pre-stretch values and low values of the normalized systolic pressure. Moreover, the peak values of the circumferential elongation (Eq.(13)), normalized thickness (Eq.(14)), normalized strain-energy density, normalized circumferential stress, and normalized longitudinal stress are investigated by varying the pre-stretch value λ_Z_^0 ^, the material parameter *b*, and the normalized systolic pressure *p_s_R / aH*. The latter response quantities are obtained on normalizing Eqs. (18), (19), and (20) by material parameter *a*.

The normalized strain energy *W / a* and the circumferential elongation of the artery are decreased with increasing values of the longitudinal pre-stretch *λ_z_^0 ^* or the material parameter *b*. Note that for the different values of material parameter *b* the calculated stresses present intersection points. Consequently, the hoop stress is not a representative criterion to obtain the response limits of different arterial systems. On the contrary, the strain-energy density and the radial displacement of different arterial systems appear distinctive (Table **2**). Volokh [32] was the first to indicate that the strain-energy density constitutes a trustworthy criterion for the arterial strength.

The normalized strain energy *W / a*, the circumferential elongation, and the normalized circumferential stress are decreased with increasing values of longitudinal pre-stretch $\lambda_{z}^{0}$. In all cases, the peak value of *W / a* occurs during the beginning of the loading.

Regarding the Mooney-Rivlin model we observe the followings. As can be seen from Figs. (**10**) and (**11**), an increase of the longitudinal pre-stretch $\lambda_{z}^{0}$ , results in a decrease of the radial displacement and in stiffer arterial systems (Fig. **10a**). On the other hand, by increasing the material parameter *β* (Fig. **10b**), the normalized systolic pressure *p_s_R/µH* (Fig. **11a**), or the normalized characteristic time *t_cp_ / t_MR_* (Fig. **11b**) , the normalized radial displacement is increased. Zero-th order resonant frequency *ω_ 0_^MR^*: (a) increases slightly fom 1000 s^-1^ to 1200 s^-1^ with $\lambda_{z}^{0}$ from 1 to 1.3 (*β = *0, *p_s_R / µH* = 0.64,* t_cp_ / t_MR_ =* 1000) (b) decreases slightly from 1250 s^-1^ to 1000 s^-1^ with *β* from -0.5 to 0.5 $\left(\lambda_{z}^{0}=1.1,p_{s}R/\mu H=0.64,t_{\mathrm{cp}}/t_{\mathrm{MR}}=1000\right)$ .The linear model yields lower radial displacements than the Mooney-Rivlin arterial model, especially for low pre-stretch values or high normalized systolic-pressure values. Note that, when the material parameter *β* is increased the effect of the second invariant is reduced, resulting in softer systems. For $\lambda_{z}^{0}$ = 1, there is no effect of the parameter *β* on the response of any system.

As follows from Fig. (**12**), the response parameters *λ_θ_*, *W / µ* and *σ_θθ_*/ µ, are increased for increasing values of the material parameter *β* whereas all response quantities are increased for increasing values of the normalized systolic pressure *p_s_R / µH*. The abrupt pressure loading gives periodic (thus bounded) radial displacement, provided *p_s_R / µH < *1 . As *p_s_R / µH →*1, the radial displacements become unbounded *u_r_* / *R* →∞, as seen in Fig. (**11a**) [33]. This could render aneurysmatic arteries vulnerable as shear modulus and thickness reduces, and artery diameter increases.

The response spectra were investigated for different values of longitudinal pre-stretch, and for three values of the material parameter *β*. As expected, the circumferential elongation is reduced with increasing values of longitudinal pre-stretch $\lambda_{z}^{0}$. It was observed that the normalized strain energy has an optimized (minimum) point corresponding to a particular pre-stretch value of 1.1 (Table **4**). Interestingly, 1.1 is the prestretch value reported *in vivo* arteries. On the other hand, the response quantities are increased with increasing values of the material parameter *β*.

Response spectra were also constructed for different longitudinal pre-stretch values and for three values of normalized systolic pressure *p_s_R / µH*. By increasing *p_s_R / µH* (case of hypertension or low elasticity modulus) the response is increased. Furthermore, for each case of normalized pressure, the normalized strain energy exhibits an optimized (minimum) point for a particular pre-stretch value (Table **5**).

Inherent limitations of the model are associated with the assumptions related to the single homogenized layer and isotropic material. However, longitudinal transverse isotropy will not alter the present results, except for the material constants [34]. In fact, several hyperelastic constitutive laws that consider more detailed arterial structure are available in the literature. The drawback of these models is that they depend on a plethora of material parameters (as opposed to the models analyzed herein, which contained only two material parameters), which cannot be easily obtained, nor are they available in the existing literature. The arterial material parameters are characterized by large uncertainty and vary with topology, age, gender, and disease of the artery. Accordingly, at this point, it may not be useful to consider more detailed multi-parameter hyperelastic laws. Viscoelasticity is yet one additional aspect that was not examined in this work. Due to its additional complexity, we have addressed the problem in a separate work [35]. It suffices to mention that without the present results a large-deformation hyperelastic formulation is pointless.

**Fig. (4):**
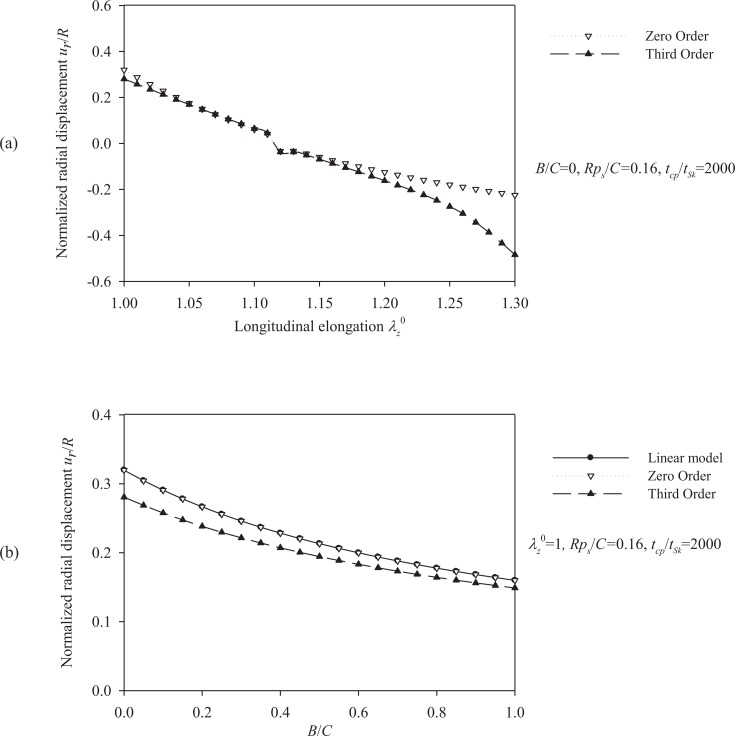
Displacement spectra of the healthy (Skalak) arterial model. Plots for **(a)** pre-stretch values λ_Z_^ 0^ = {1 ÷ 1.3} and *B/C* = 0, *p_s_R/C* = 0.16, *t_cp_/t_Sk_* = 2000, **(b)** ratios *B/C*= {0 ÷ 1} and *λ_Z_^0 ^* = 1, *p_s_R/C* = 0.16, *t_cp_ / t_Sk_* = 2000.

**Fig. (5):**
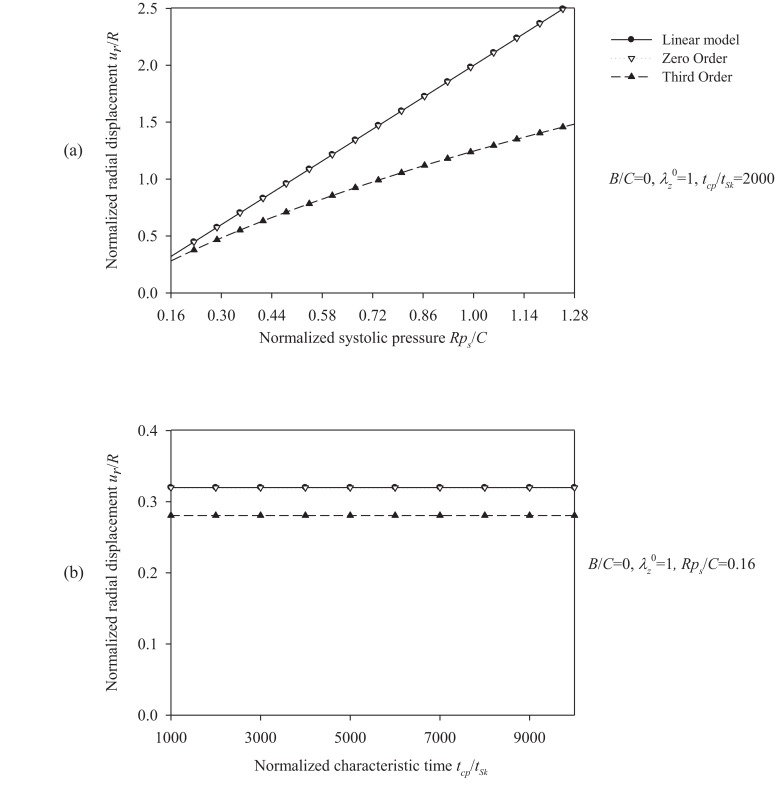
Displacement spectra of the healthy (Skalak) arterial model. Plots for **(a)** normalized systolic pressure values *p_s_R / C* = {0.16 ÷ 1.28} and *B / C* = 0, $\lambda_{z}^{0}$ = 1, *t_cp_/t_Sk_* = 2000, **(b)** normalized characteristic time values *t_cp_/t_Sk_* = {1000 ÷ 10000} and *B /C =* 0,* λ_Z_^ 0^* = 1, *p_s_R/C* = 0.16.

In order to establish a basis of comparison between the linear-elastic and hyperelastic displacement response, the same initial tangent Young's modulus derived from the circumferential Cauchy stress-strain relationship was used. It appears that, in most cases, the solution of the linear model is conservative compared to the Skalak model, constituting overall a good approximation of the Skalak solution (Figs. **4** and **5**). The Mooney-Rivlin model yields larger radial displacements compared to the linear model (Figs. **10** and **11**), as expected. On the other hand, the linear approximation is not good for the Hariton model, especially for low pre-stretch values, due to the fact that the initial tangent modulus approaches zero (the slope of the stress-strain curve becomes steeper at higher strains, (Figs. **7** and **8**). The use of the tangent Young's modulus corresponding to circumferential strains between 10%-20% is expected to yield better approximations.

As demonstrated in this study, the peak response of the hyperelastic models is heavily influenced by the longitudinal pre-stretch $\lambda_{z}^{0}$ and the normalized pressure value. In particular, the normalized radial displacement decreases with increasing values of pre-stretch (Figs. **4a**, **7a** and **10a**). Fig. (**14**) offers a reasoning as to what this means for the human health along the years, for the case that the material law is not significantly altered over the years. The longitudinal pre-stretch is caused due to the delayed growth of arteries compared to the rest of the body.

Therefore, human arteries exhibit increasing longitudinal pre-stretch with aging. The gradual arterial stress softening, caused by aging, can be balanced by the longitudinal pre-stretch and the decreased radial response. On the other hand, at old age the human body exhibits small shrinkage causing the longitudinal pre-stretch to decrease. Combined with the continuous loss of strength, the arterial response cannot be easily balanced, thus the human vascular system becomes vulnerable.

In cases of hypertension or soft arterial tissue, the normalized pressure is increased. Increasing the normalized pressure results in an amplified artery displacement and stressing (Figs. **5a**, **8a**, **11a**), especially for the Mooney-Rivlin model as the systolic pressure approaches *µH / R*. This indicates a limit index between artery radius, artery thickness, shear modulus and systolic pressure. Aneurysms could become critical if *p_s_R / µH* → 1.

Of particular interest is the magnitude of the strain-energy density of the arterial systems. The normalized strain energy is increased with increasing values of the absolute normalized displacement $\lambda_{z}^{0}$.

**Fig. (6):**
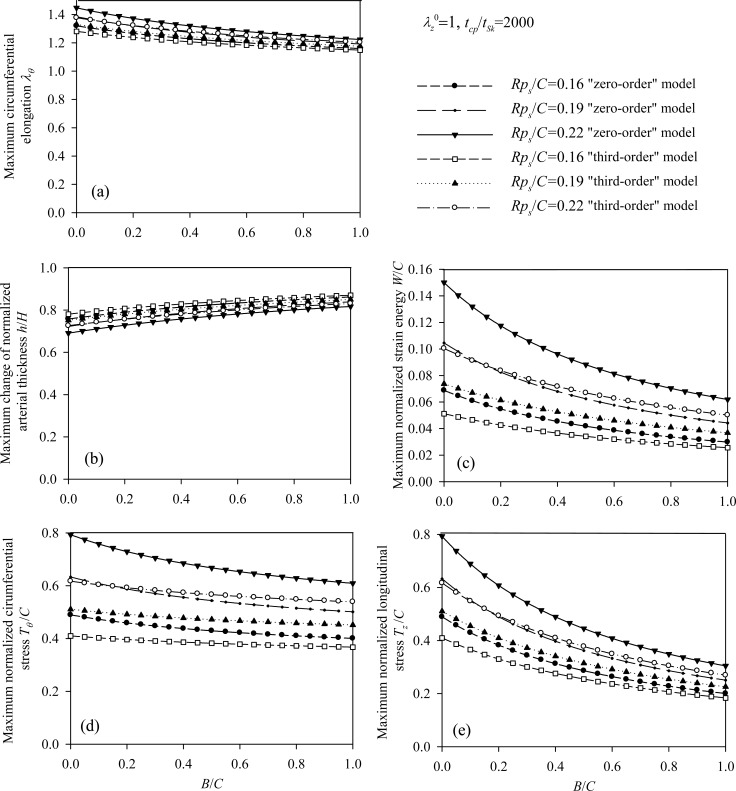
Response spectra of the healthy (Skalak) arterial model. Plots for *B / C = *{0 ÷ 1}, for three values of the normalized systolic pressure *p_s_R/C* and for *λ_Z_^ 0^ =* 1,* t_cp_ / t_sk_* = 2000: **(a)** circumferential elongation λ_θ_, **(b)** normalized thickness *h/H*, **(c)** normalized strain energy *W/C*, **(d)** normalized circumferential stress *T_ θ_ / C*, **(e)** normalized longitudinal stress *T_z_ / C*.

**Fig. (7):**
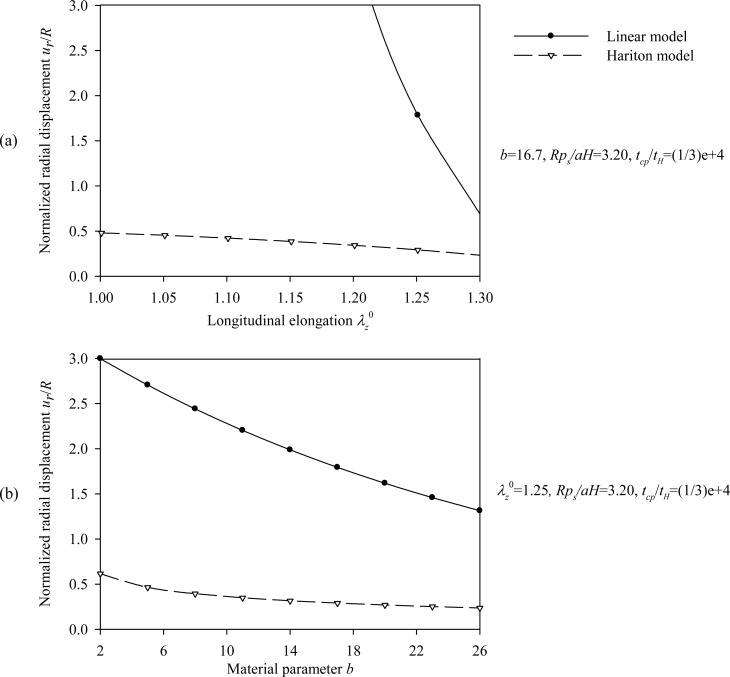
Displacement spectra of the atherosclerotic (Hariton) arterial model. Plots for **(a)** pre-stretch values λ_z_^0^ {1 ÷ 1.3} and *b* = 16.7, *p_s_R/aH = *3.2,* t_cp_ / t_H_* = 1 / 3E-4, **(b)** material parameter values *b = *{2 ÷ 26} and *λ_z_^0 ^* = 1.25, *p_s_R/C = *3.2,* t_cp_ / t_H_* = 1 / 3E-4.

**Fig. (8):**
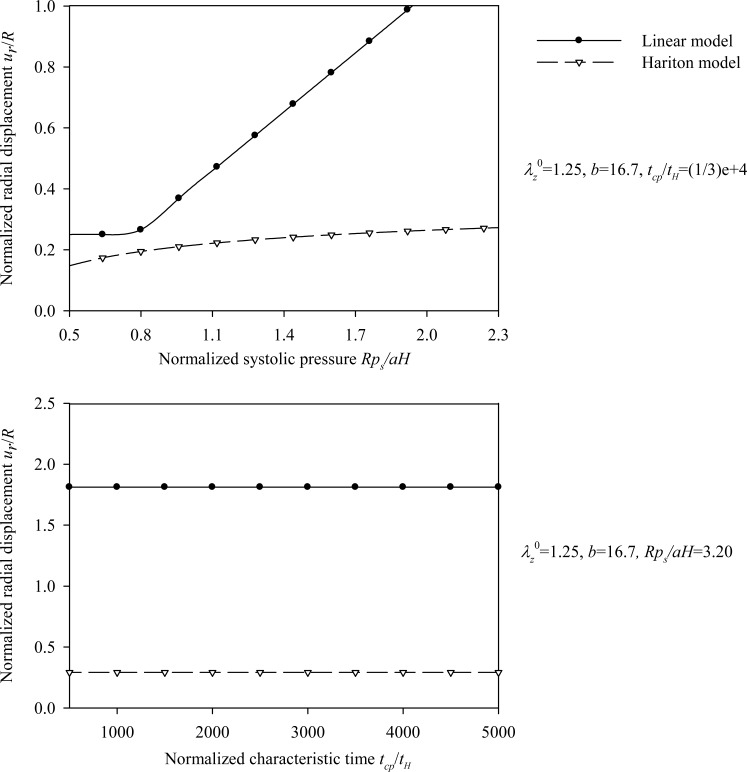
Displacement spectra of the atherosclerotic (Hariton) arterial model. Plots for **(a)** normalized systolic pressure values *p_s_R/aH =*{0.5 ÷ 2.3} and*  *λ_z_^0^ = 1.25,  *b* = 16.7*,* *t_cp_ / t_H_* = 1 / 3E-4. and , **(b)** normalized characteristic time values *t_cp_/ t_H_* = {500 ÷ 5000} and λ_z_^0^ = 1.25, b=16.7, *p_s_R/aH* = 3.2.

**Fig. (9):**
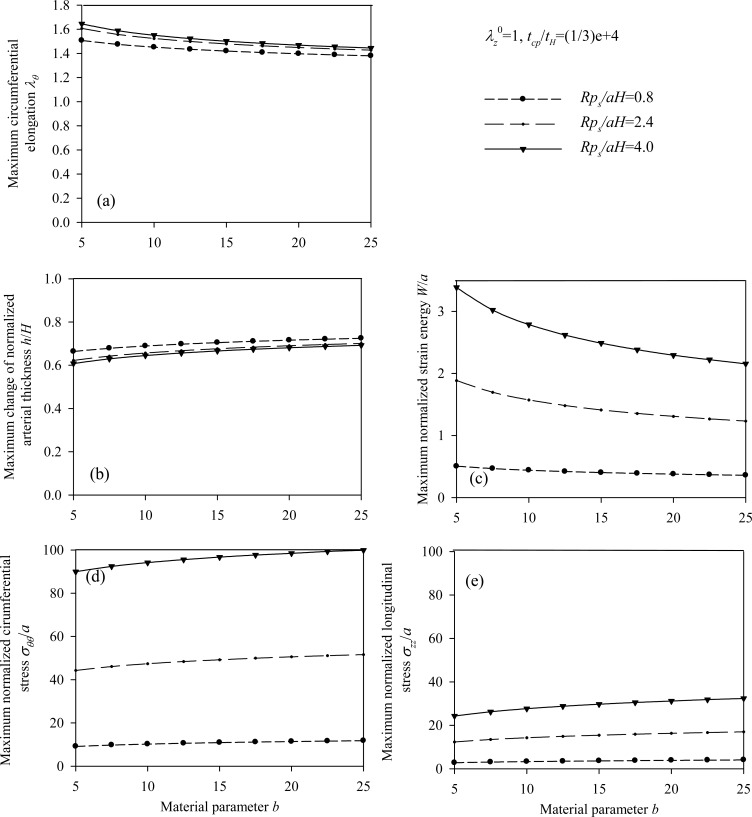
Response spectra of the atherosclerotic (Hariton) arterial model. Plots for *b* = {5 ÷ 25}, for three values of the normalized systolic pressure *p_s_R / aH* and for *λ_z_^ 0^ = *1,* t_cp_/t_H_ =* 1/3E - 4: **(a)** circumferential elongation λ_θ_, **(b)** normalized thickness *h / H*, **(c)** normalized strain energy *W / a,* **(d)** normalized circumferential stress σ_θθ_/ *a*, **(e)** normalized longitudinal stress σ_ZZ_/*a*.

**Fig. (10):**
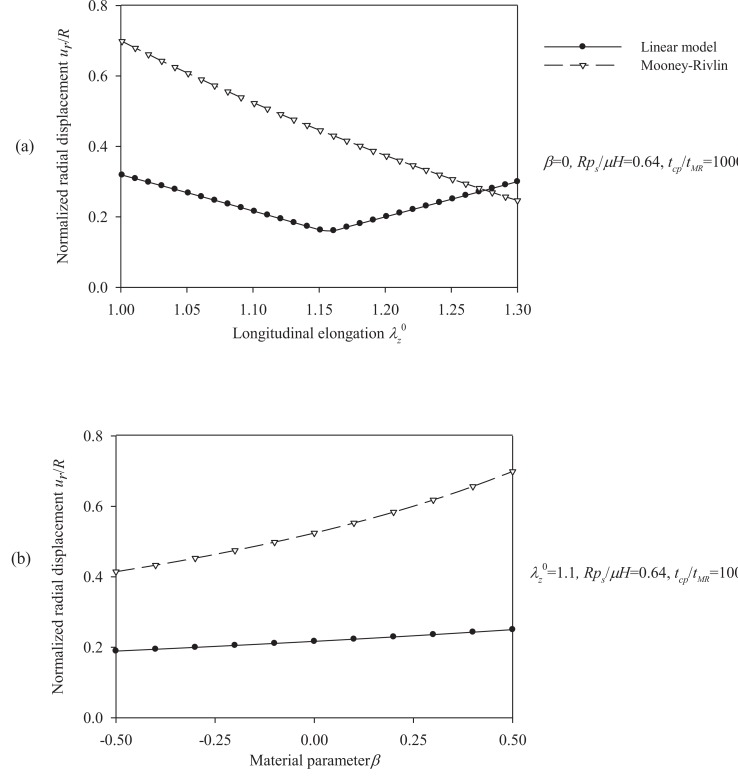
Displacement spectra of the aneurysmatic (Mooney-Rivlin) arterial model. Plots for **(a)** pre-stretch values λ_z_^ 0^ = {1 ÷ 1.3} and *β* = 0, *p_s_R/µH* = 0.64, *t_cp_ / t_MR_* = 1000, **(b)** material parameter values *β* = {-0.5 ÷ 0.5} and λ_z_^0^ = 1.1, *p_s_R/µH* = 0.64, *t_cp_ / t_MR_* = 1000.

**Fig. (11):**
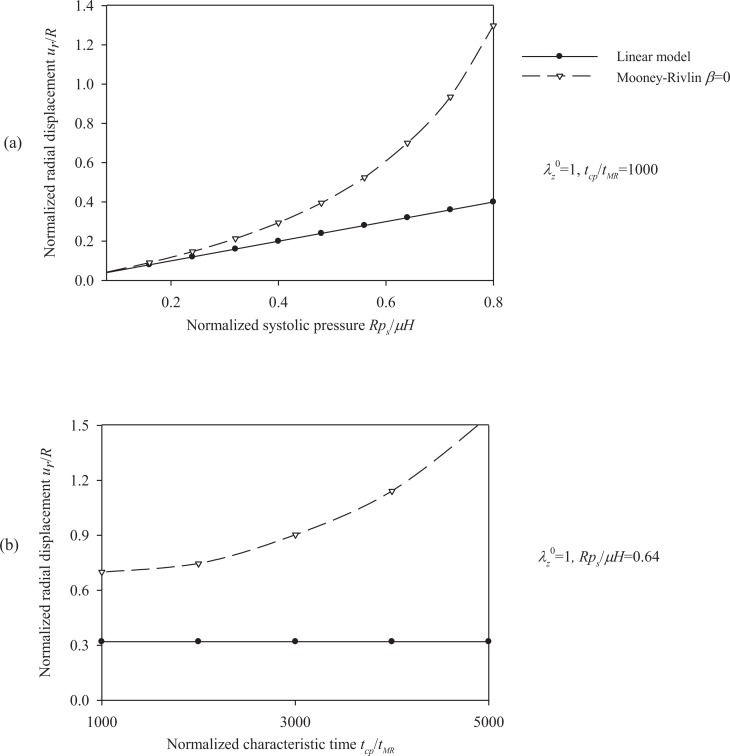
Displacement spectra of the aneurysmatic (Mooney-Rivlin) arterial model. Plots for **(a)** normalized systolic pressure values psR/µH ={0.08 ÷ 0.8} and λ_z_^0^ = 1, β = 0,* t_cp_ / t_MR_* = 1000, **(b)** characteristic time values *t_cp_ / t_MR_* = {1000 ÷ 5000} and *λ_z_^ 0^ = *1,* β = *0,* p_s_R/ µH = *0.64.

**Fig. (12):**
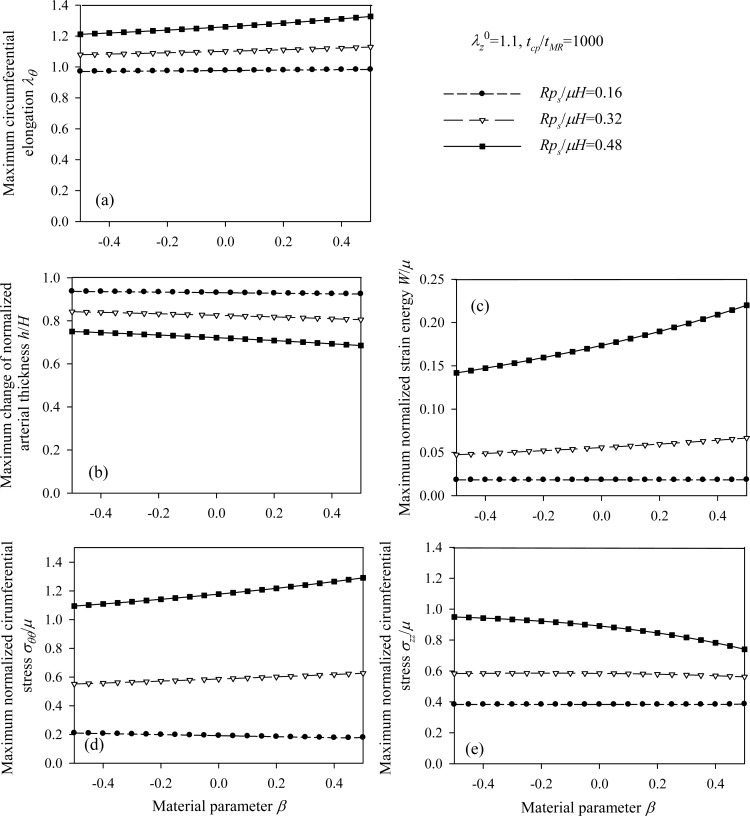
Response spectra of the aneurysmatic (Mooney-Rivlin) arterial model. Plots for *β* = {-0.5 ÷ 0.5}, for three values of the normalized systolic pressure *p_s_R/ µH* and for λ_z_^0^ = 1.1, *t_cp_ / t_MR_* = 1000: **(a)** circumferential elongation *λ_θ_*, **(b)** normalized thickness *h / H*, **(c)** normalized strain energy *W / µ*, **(d)** normalized circumferential stress *σ_θθ_/µ*, **(e)** normalized longitudinal stress *σ_ZZ_/µ*.

**Fig. (13):**
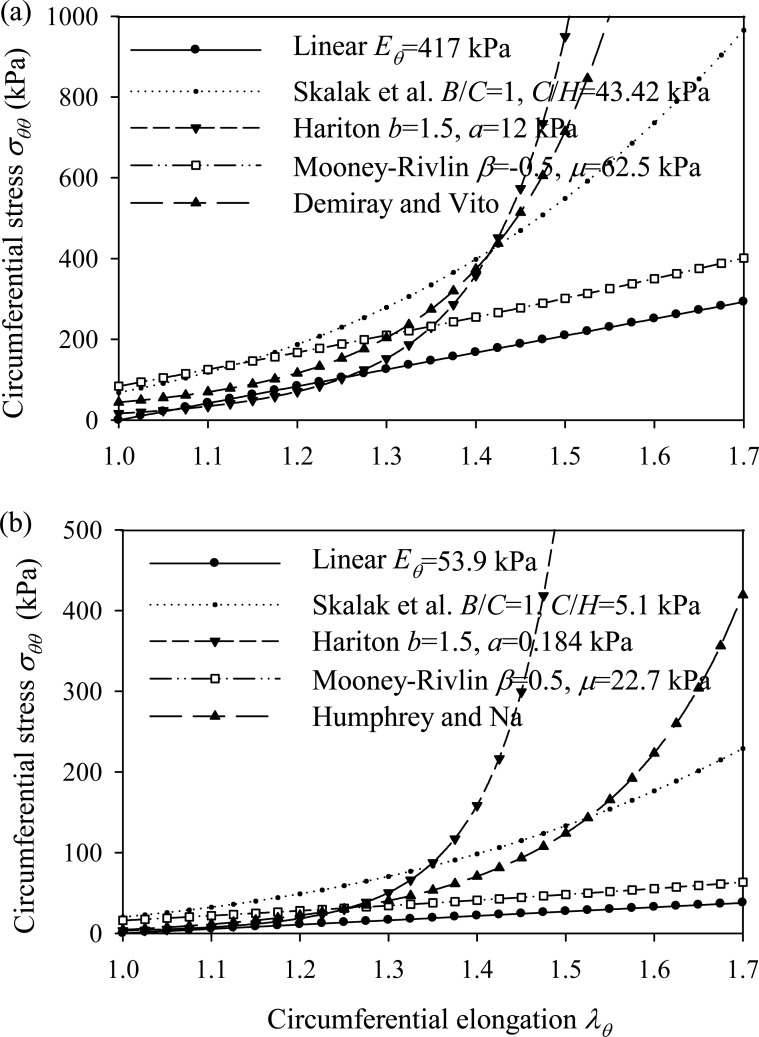
Circumferential stress-strain curves of the linear and hyperelastic constitutive laws used in the numerical examples. Based on the data of **(a)** Demiray and Vito [15] **(b)** Humphrey and Na [13].

In some cases, the stress value is not an appropriate criterion for distinguishing the limit strength of different arterial systems. On the contrary, the corresponding strain energies and displacements are distinctive, making such quantities more trustworthy criteria for healthy arterial response (Figs. **6**, **9**, **12**, and Tables **1**-**5**). The energy density criterion of the arterial tissue is consistent with a failure criterion, namely, if the energy density of the system reaches a limit value the artery will fail.

It was suggested that post-stenotic weakening of arterial walls is caused by arterial wall vibrations and structural fatigue initiated by pressure disturbances in turbulent blood flow. Therefore, dynamic effects can be very important and resonant frequencies can be triggered by abrupt application of the blood pressure as suggested in this work. High-frequency vibration generated by turbulence downstream from stenosis appears to be transmitted to the upstream wall and increase the possibility of rupture at atherosclerotic plaque, or destabilize aneurysmatic arteries [36]. New applications can also emerge from this work regarding the assessment of the dynamic elastance of superficial arterial walls (see for example, Wang *et al*. [37]).

### 4.1 Connection with Arterial Pressure Wave Propagation

A recent analysis of arterial wave propagation along the artery using direct measurements of blood velocity and arterial wall diameter has been undertaken by Feng and Khir [38].

The wave speed *c* along the arterial tube is given by

(31) $$c^{2}=\frac{A}{\rho_{b}}\frac{\mathrm{dP}}{\mathrm{dA}}$$

which is a relation between the cross sectional area *A* of the artery and the local transmural pressure *P* (*ρ_b_* is the blood density). Using the wavelet approach of Parker and co-workers [39, 40] one can cast the wave speed as

(32) $$c^{2}=\pm \frac{1}{2}\frac{\mathrm{dU}}{d\left({\mathrm{lnD}}_{\pm }\right)}$$

where *U* is the local average blood velocity and *D=2(R+u_r_)* is the local artery diameter (± denote pressure pulses in the forward and backward directions). Feng and Khir [38] measured simultaneously *D* and *U* waveforms and obtained c≈6.9m/s for human carotid artery. A close look at the results of Feng and Khir show high frequency wave upon long waves for *D(t)* wave forms. Such waves are also shown by Meinders and Hoeks [41] and Canic *et al*. [42], which of course, are due to the radial wall vibrations. Moreover, Feng and Khir confirmed that In *D* and *U* are linearly related in the absence of wave reflections, as also indicated by Womersley [43] for the linear elastic response of arteries. The technique of wavelets bypasses the need for pressure measurements and uses the diameter waveform, irrespective of the relationship between pressure and diameter.

Meinders *et al*. [44] proposed measurement of the arterial diameter waveforms directly at certain positions along the artery by means of ultrasound imaging (having the advantage of being non-invasive). Thereafter, the diameter information is converted to pressure *p* using an empirically derived exponential relation, Meinders and Hoeks [41].

(33) $$A\left(t\right)=\pi D^{2}\left(t\right)/4$$

(34) $$p\left(t\right)=p_{d}\;\exp\left(\frac{\alpha A\left(t\right)}{A_{d}}-1\right)$$

where *p_d_* is the diastolic pressure, *A_d_* is the diastolic arterial cross section and *α* is a positive dimensionless parameter that depends on the artery properties. Therefore, by inversion one can obtain *A(p)*, that is the area as a function of pressure, as a local valve along the specific locations of the artery that *D(t)* is measured. The parameter *a* was measured from 1 to 8 and increases with age (in years) for carotid arteries approximately as

(35) α=0.421+0.0602×age

Meinders *et al*. [44] measured multiple adjacent diameter distension waveforms as

(36) $$\frac{1}{A\left(p\right)}\frac{\mathrm{dA}\left(p\right)}{\mathrm{dp}}=\frac{1}{\rho_{b}c^{2}}$$

simultaneously along a short carotid artery and found *c ≈ *5.5m/s for young healthy subjects.

Turning to our analysis, we can easily show that our method can provide the stretching for the systolic and diastolic pressures *p_s_* and *p_d_* denoted by *λ_θ_^s^* and *λ_θ_^d^* accordingly. Remembering that *λ_θ_^s^* = 1 + *u_r_^s^ / R* and *λ_θ_^d^* = 1 + *u_r_^d^ / R*, we can obtain that

(37) $$\alpha =\frac{\ln\left(p_{s}/p_{d}\right)}{{\left(\lambda_{\theta }^{s}/\lambda_{\theta }^{d}\right)}^{2}-1}$$

The following Table **8** gives estimates for the *α* parameter in accord with the experiments.

A few words should be mentioned regarding the axial displacement *u_z_* that has been recently found to be important, see for example Cinthio *et al*. [45]. We can follow the general approach of Naghdi and Cooper [46] to estimate the relative magnitude of the systolic axial displacement $\left|u_{z}^{s}/u_{r}^{s}\right|$ relative to the systolic radial displacement. To this end, we need the Poisson ratio (*v* = 1/2), the thickness of the artery (*H* = 0.6 mm), the radius of the artery (*R* = 3 mm), the ratio of the artery density to the blood density (*ρ_0 _ / ρ_b_ ≈* 1) the longitudinal wave length (λ* ≈ * 120 mm), and the phase velocity (c* ≈* 5m/s). The natural frequency of the surrounding tissue is 2πc /λ ≈ 262rad/s. Using the linear elastic approximation for the arterial wall

(38) $$\left|\frac{u_{z}^{s}}{u_{r}^{s}}\right|\approx \frac{v\frac{\lambda }{2\pi R}}{1-\frac{c^{2}}{c_{p}^{2}}}$$

with $c_{p}^{2}=E_{\theta }^{0}/\left(1-v^{2}\right)\rho_{0}$.

The result is $\left|u_{z}^{s}/u_{r}^{s}\right|$ ≈ 3.2 which together with *u_r_^s^* = 0.29mm gives $\left|u_{z}^{s}\right|\approx 0.92$ mm, close to the 0.9 mm value of Cinthio *et al*. [45] (see also Warriner *et al*. [47]).

## CONCLUSION

The present study proposes an analytical method to investigate the strain-hardening effect on the dynamic behavior of human artery segments. The governing equation of motion has been formulated accounting for three different hyperelastic material behaviors: (a) the constitutive law proposed by Skalak *et al.*, associated with the hardening behavior of healthy arteries; (b) the constitutive law introduced by Hariton, associated with the hardening behavior of atherosclerotic arteries; and (c) the constitutive law of Mooney-Rivlin, associated with the softening behavior of aneurysmatic arteries.

The response of each model was numerically investigated calculating in a general manner the radial displacement, circumferential elongation, circumferential stress, the longitudinal stress, and the strain-energy density. The analysis showed that the peak response of the hyperelastic models is strongly affected by the longitudinal pre-stretch and the normalized pressure. In particular, a decrease in the pre-stretch and/or an increase in the normalized pressure (case of hypertension or soft arterial tissue) results in an increase in the normalized radial displacement. Of particular interest for the stability of artery response, is the assessment of the natural frequencies of the various models, especially for the softening models like the Mooney-Rivlin that models aneurysmatic arteries.

As demonstrated in this study, important metrics that can be useful to vascular analysis are the radial deformation and the maximum strain-energy density. These metrics were found to be influenced heavily by the strain-hardening characteristics of the model and the longitudinal pre-stressing. It has been shown that, the strain-energy density is directly related to the normalized radial displacement, $\left|u_{r}/R\right|$, (with the strain-energy density being increased with increasing values of the absolute normalized radial displacement), thus making the strain-energy density a trustworthy criterion for the arterial strength.

The analytical formulation of the problem permits a systematic and generalizable investigation of the hyperelastic models commonly used in the mechanical modeling of arteries, which, together with the low computational cost of analysis, makes the proposed model a valuable tool for calculating the response of healthy, atherosclerotic, and aneurysmatic artery segments. Our model can be used for the natural biomechanical test of a cyclic inflation of a straight artery segment. The model can easily adapt for the different mechanical properties encountered along a real artery. The present results are useful in the development of circulatory system models providing the time evolution of the cross-sectional area $\left(\pi \left(R+u_{r}{\left(t\right)}^{2}\right)\right)$ that is required by fluid-solid interaction models. The present results are also prerequisites for the development of more complex models that include viscosity [35].

## Figures and Tables

**Fig. (1):**
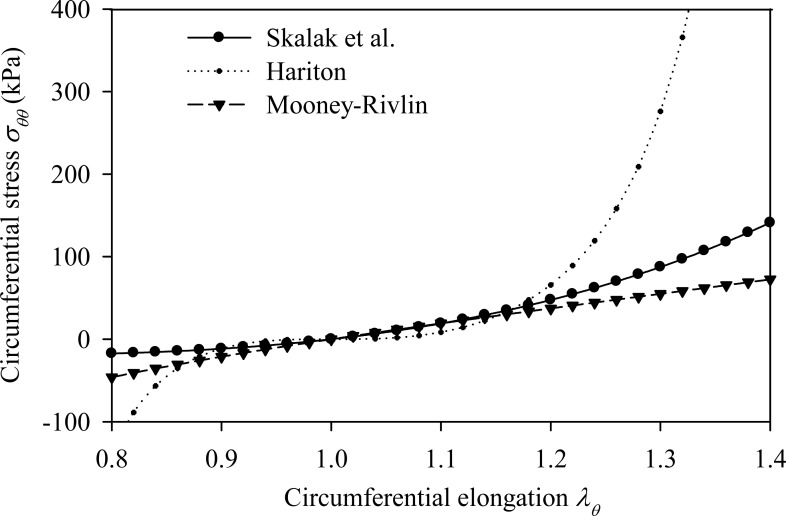
Stress-strain diagrams of hyperelastic incompressible models. No pre-stretch is applied to the models $\left(\lambda_{z}^{0}=1\right)$. By *σ_θθ_* is denoted the circumferential Cauchy stress.

**Fig. (2):**
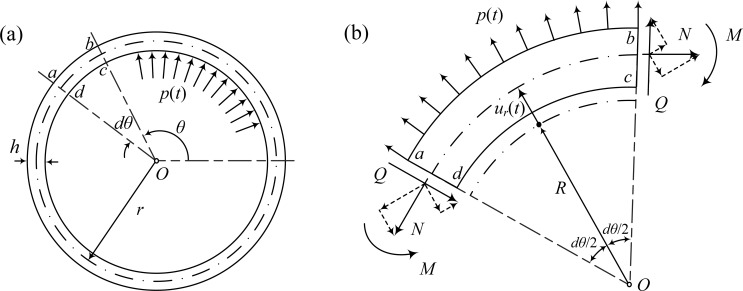
Proposed arterial model. **(a)** Arterial model at deformed state, **(b)** typical element of circular ring at deformed state.

**Fig. (3):**
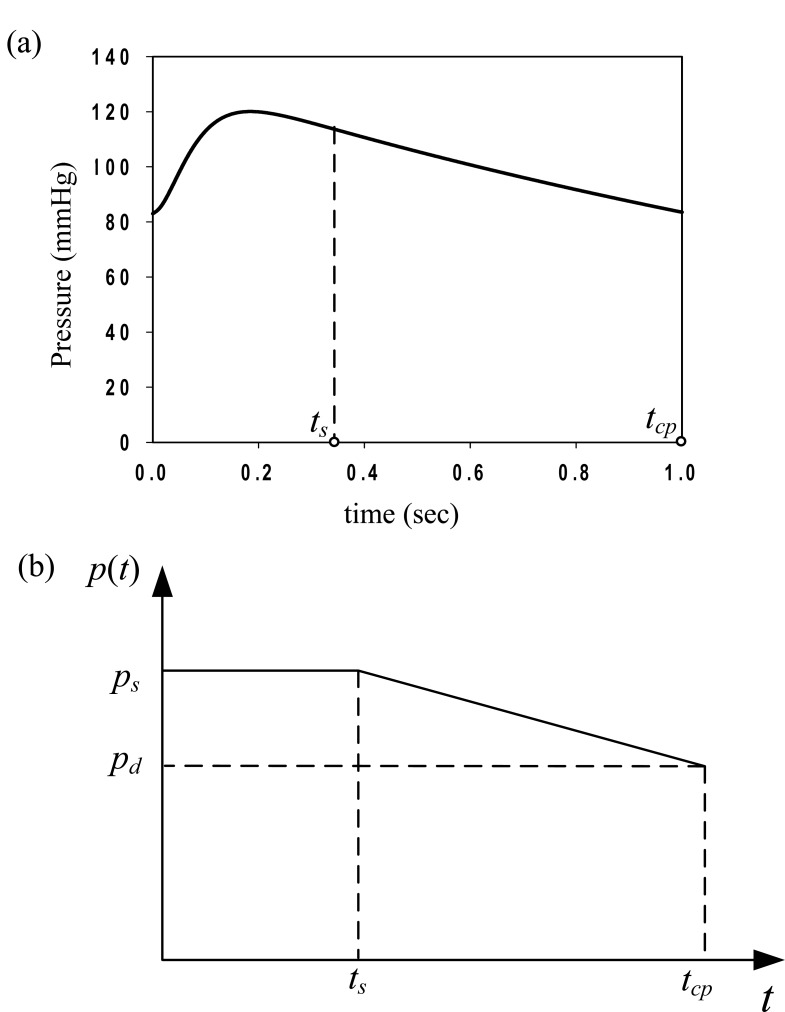
Blood pressure time-profiles approximations (after Roussis *et al*. [23, 17]): **(a)** Aortic pressure time-profile following Zhong *et al*. [22, 16], **(b)** arterial pulse time-profile approximation.

**Fig. (14):**
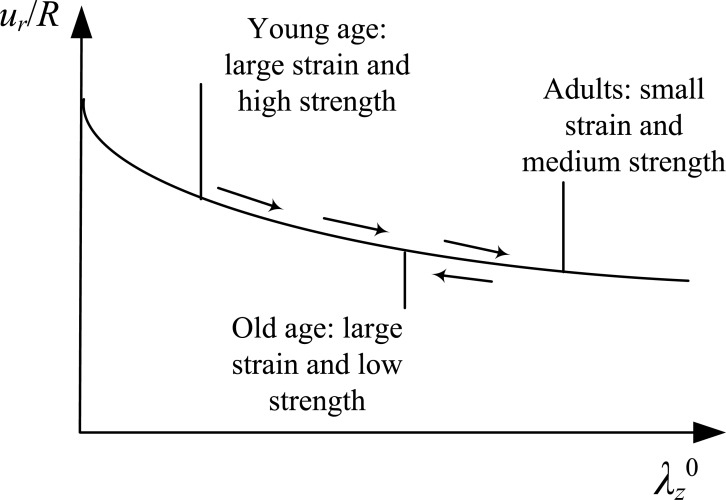
Radial deformation as a function of the longitudinal pre-stretch. Explanatory diagram for the longitudinal pre-stretch and radial deformation effect on the arterial behavior of different age groups. It is assumed that the material law is not significantly changed over the years.

**Table 1:** Maximum normalized strain energy *W/C* and occurrence time for range of λ_z_^ 0^ and *B / C* values (*p_s_R/C = *0.16).

λ_z_^ 0^	*B/C*					
	0		0.5		1	
	Maximum *W/C*	Time (sec)	Maximum *W/C*	Time (sec)	Maximum *W/C*	Time (sec)
1	0.051184	0.001383	0.034112	0.001174	0.025579	0.00104
1.05	0.0321	0.001349	0.02378	0.001163	0.019512	0.001038
1.1	0.017248	0.001319	0.017016	0.001154	0.017996	0.001039
1.15	0.013001	0	0.019501	0	0.026002	0
1.2	0.0242	0	0.0363	0	0.0484	0
1.25	0.041494	0.35078	0.060049	0.35011	0.079324	0.34928
1.3	0.097124	0.99934	0.10226	0.99997	0.12222	0.35024

**Table 2:** Maximum normalized strain energy *W / a* and occurrence time for range of λ_z_^0^ and *b* values (*p_s_R / aH* = 3.2).

λ_z_^0^	*b*					
	5		15		25	
	Maximum *W / a*	Time (sec)	Maximum *W / a*	Time (sec)	Maximum *W / a*	Time (sec)
1	2.6255	0.00020	1.9443	0.00017	1.6900	0.00016
1.05	2.6272	0.00019	1.913	0.00017	1.6495	0.00016
1.1	2.5968	0.00019	1.8488	0.00016	1.5688	0.00015
1.15	2.5317	0.00019	1.747	0.00016	1.4549	0.00015
1.2	2.4317	0.0009	1.6072	0.00016	1.2991	0.00014
1.25	2.2964	0.00018	1.4287	0.00015	1.0992	0.00014
1.3	2.1241	0.00018	1.2076	0.00015	0.8538	0.00013

**Table 3:** Maximum normalized strain energy *W / a* and occurrence time for range of *λ_z_^0 ^* and *p_s_R / aH* values (*b* = 15).

λ_z_^0^	*p_s_R / aH*					
	0.8		2.4		4	
	Maximum *W / a*	Time (sec)	Maximum *W / a*	Time (sec)	Maximum *W / a*	Time (sec)
1	0.40285	0.00033	1.4107	0.00020	2.4879	0.00015
1.05	0.39027	0.00032	1.3863	0.00019	2.4441	0.00015
1.1	0.36872	0.00032	1.3341	0.00019	2.374	0.00019
1.15	0.33763	0.00031	1.2558	0.00018	2.2506	0.00014
1.2	0.29761	0.00030	1.1483	0.00018	2.0825	0.00014
1.25	0.2494	0.00030	1.0106	0.00017	1.8603	0.00013
1.3	0.19548	0.00029	0.84355	0.00017	1.5873	0.00013

**Table 4:** Maximum normalized strain energy *W / µ* and occurrence time for range of λ_z_^0^ and *β* values (*p_s_R / µH* = 0.32).

λ_z_^0^	*β*					
	-0.5		0		0.5	
	Maximum *W / µ*	Time (sec)	Maximum *W / µ*	Time (sec)	Maximum *W / µ*	Time (sec)
1	0.0757	0.34535	0.0757	0.34535	0.0757	0.34535
1.05	0.0558	0.001836	0.0606	0.001897	0.0662	0.31852
1.1	0.0473	0.001788	0.0556	0.001902	0.0666	0.002042
1.15	0.048	0.001739	0.0589	0.001902	0.0754	0.002115
1.2	0.0672	0	0.0697	0.005671	0.0916	0.002191
1.25	0.1013	0	0.1013	0	0.1145	0.002283
1.3	0.1409	0	0.1409	0	0.1435	0.002305

**Table 5:** Maximum normalized strain energy *W / µ* and occurrence time for range of *λ_z_^ 0^* and *p_s_R / µH* values (β = 0).

λ_z_^0^	*p_s_R / µH*					
	0.16		0.32		0.48	
	Maximum *W / µ*	Time (sec)	Maximum *W / µ*	Time (sec)	Maximum *W / µ*	Time (sec)
1	0.0152	0.001724	0.0757	0.34535	0.2298	0.35121
1.05	0.0114	0.005146	0.0606	0.001897	0.1958	0.35078
1.1	0.0182	0	0.0556	0.001902	0.1735	0.34967
1.15	0.0393	0	0.0589	0.001902	0.1612	0.34799
1.2	0.0672	0	0.0697	0.005671	0.1578	0.002133
1.25	0.1013	0	0.1013	0	0.1621	0.002123
1.3	0.1409	0	0.1409	0	0.173	0.002116

**Table 6:** Data and response values of the Demiray and Vito [15] numerical example. Data based on the Demiray and Vito study, and parameters and response values of the respective arterial models proposed in this study.

Data	
*R* (mm)	3.46
*H* (mm)	0.68
λ_z_^0^	1.53
*ρ_0 _* (kg/m^3^)	1160
*p_s_* (mmHg) /(Pa)	74.2/9892
*p_d_* (mmHg) /(Pa)	26.0/3466
*t_s_* (sec)	0.35
*t_cp_* (sec)	1
Linear arterial model	
*Parameters*	
*E_θ_*(kPa)	4.17
*u_0 _* (mm)	1.83
*Peak response values*	
*u_r_ / R*	0.53
*σ_θθ_* (kPa)	221
*σ_zz_* (kPa)	221
*W* (kPa)	117
Skalak arterial model	
*Dimensionless parameters*	
*B / C*	1
*p_s_R / C*	1.16
*t_cp_ / t_Sk_*	2187
*Peak response values*	
*u_r_ / R*	0.06
*σ_θθ_* (kPa)	96.68
*σ_zz_* (kPa)	161.75
*W* (kPa)	39.56
Hariton arterial model	
*Dimensionless parameters*	
*b*	1.5
*p_s_R / aH*	4.19
*t_cp_ / t_H_*	1143
*Peak response values*	
*u_r_ / R*	0.42
*σ_θθ_* (kPa)	446.69
*σ_zz_* (kPa)	523.77
*W* (kPa)	43.90

**Table 7:** Data and response values of the Humphrey and Na [13] numerical example. Data based on the Humphrey and Na study, and parameters and response values of the respective arterial models proposed in this study.

Data	
*R* (mm)	1.69
*H* (mm)	0.6
λ*_z_^ 0^*	1.832
*ρ_0 _* (kg/m^3^)	1160
*p_s_* (mmHg) /(Pa)	55/ 7.333
*p_d_* (mmHg) /(Pa)	41/5.466
*t_s_* (sec)	0.3
*t_cp_* (sec)	0.8
Linear arterial model	
*Parameters*	
*E_θ_* (kPa)	53.9
*u_0 _* (mm)	1.406
*Peak response values*	
*u_r_ / R*	0.83
*σ_θθ_* (kPa)	44.85
*σ_zz_* (kPa)	44.85
*W* (kPa)	37.31
Skalak arterial model	
*Dimensionless parameters*	
*B / C*	0.5
*p_s_R / C*	4.04
*t_cp_ / t_Sk_*	1679
*Peak response values*	
*u_r_* / *R*	0.55
*σ_θθ_* (kPa)	153.22
*σ_zz_* (kPa)	164.83
*W* (kPa)	103.43
Hariton arterial model	
*Dimensionless parameters*	
*b*	1.5
*p_s_R / aH*	111.9
*t_cp_ / t_H_*	319
*Peak response values*	
*u_r_* / *R*	0.46
*σ_θθ_* (kPa)	354.27
*σ_zz_* (kPa)	569.67
*W* (kPa)	22.28

**Table 8:** Estimation of *α* parameter (*p_s_* = 16kPa, *p_s_/p_d_* = 1.51, *R = *3.38 mm,* H* = 0.6*mm*).

*p_s_R / C*	0.80	0.16	0.16
*B / C*	0.5 (soft)	0.5 (medium stiff)	1 (stiff)
λ_z_^0^	1.1	1	1
*u_r_^s^ / R*	0.2960	0.0972	0.0745
*u_r_^d^ / R*	0.1961	0.0644	0.04932
*α*	2.37	6.59	8.49

## References

[1] Shadwick R.E. (1999). Mechanical design in arteries.. J. Exp. Biol..

[2] Fung Y.C. (1998). Biomechanics: Motion, Flow, Stress, and Growth..

[3] Fung Y.C. (1984). Biodynamics: Circulation..

[4] Mohan D., Melvin J.W. (1983). Failure properties of passive human aortic tissue. II--Biaxial tension tests.. J. Biomech..

[5] Wertheim G. (1847). Mémoire sur l’élasticité et la cohésion des principaux tissus du corps humain.. Ann. Chim. Phys..

[6] Mooney M. (1940). A theory of large elastic deformation.. J. Appl. Phys..

[7] Chadwick P. (1972). The existence and uniqueness of solutions to two problems in the Mooney-Rivlin theory for rubber.. J. Elast..

[8] Fung Y.C. (1993). Biomechanics: Mechanical Properties of Living Tissues..

[9] Gent A.N. (1996). A new constitutive relation for rubber.. Rubber Chem. Technol..

[10] Skalak R., Tozeren A., Zarda R.P., Chien S. (1973). Strain energy function of red blood cell membranes.. Biophys. J..

[11] Delfino A., Stergiopulos N., Moore J.E., Meister J-J. (1997). Residual strain effects on the stress field in a thick wall finite element model of the human carotid bifurcation.. J. Biomech..

[12] Holzapfel G.A., Sommer G., Gasser C.T., Regitnig P. (2005). Determination of layer-specific mechanical properties of human coronary arteries with nonatherosclerotic intimal thickening and related constitutive modeling.. Am. J. Physiol. Heart Circ. Physiol..

[13] Humphrey J.D., Na S. (2002). Elastodynamics and arterial wall stress.. Ann. Biomed. Eng..

[14] Hariton I. (2007). Vascular Biomechanics: Functional Adaptation. Anisotropy and Seeds of Micromechanics, Ph.D. Thesis.

[15] Demiray H., Vito R.P. (1983). On large periodic motions of arteries.. J. Biomech..

[16] Baltgaile G. (2012). Arterial wall dynamics.. Perspectives in Medicine.

[17] Pedley T.J. (2003). Mathematical modelling of arterial fluid dynamics.. J. Eng. Math..

[18] Formaggia L., Quarteroni A., Veneziani A. (2010). Cardiovascular Mathematics-Modeling and simulation of the circulatory system..

[19] Taylor C.A., Humphrey J.D. (2009). Open problems in computational vascular biomechanics: Hemodynamics and arterial wall mechanics.. Comput. Methods Appl. Mech. Eng..

[20] David G., Humphrey J.D. (2003). Further evidence for the dynamic stability of intracranial saccular aneurysms.. J. Biomech..

[21] Foreman J.E., Hutchison K.J. (1970). Arterial wall vibration distal to stenoses in isolated arteries of dog and man.. Circ. Res..

[22] Zhong L., Ghista D.N., Ng E.Y., Lim S.T., Chua T.S. (2004). Determination of aortic pressure-time profile, along with aortic stiffness and peripheral resistance.. J. Mech. Med. Biol..

[23] Roussis P.C., Giannakopoulos A.E., Charalambous H.P., Demetriou D.C., Georghiou G.P. (2015). Dynamic behavior of suture-anastomosed arteries and implications to vascular surgery operations.. Biomed. Eng. Online.

[24] Knowles J.K. (1960). Large amplitude oscillations of a tube of incompressible elastic material.. Q. Appl. Math..

[25] Shampine L., Reichelt M. (1997). The MATLAB ODE Suite.. SIAM J. Sci. Comput..

[26] (2011). MATLAB R2011b..

[27] Shampine L.F. (1982). Implementation of rosenbrock methods.. ACM Trans. Math. Softw..

[28] Zedan H. (1990). Avoiding the exactness of the Jacobian matrix in Rosenbrock formulae.. Comput. Math. Appl..

[29] Hosea M.E., Shampine L.F. (1996). Analysis and implementation of TR-BDF2.. Appl. Numer. Math..

[30] Blatz P.J., Chu B.M., Wayland H. (1969). On the mechanical behavior of elastic animal tissue, 1957-1977. Trans. Soc. Rheol..

[31] Chuong C.J., Fung Y.C. (1983). Three-dimensional stress distribution in arteries.. J. Biomech. Eng..

[32] Volokh K.Y. (2007). Hyperelasticity with softening for modeling materials failure.. J. Mech. Phys. Solids.

[33] Shahinpoor M., Nowinski J.L. (1971). Exact solution to the problem of forced large amplitude radial oscillations of a thin hyperelastic tube.. Int. J. Non-linear Mech..

[34] Mason D.P., Maluleke G.H. (2007). Non-linear radial oscillations of a transversely isotropic hyperelastic incompressible tube.. J. Math. Anal. Appl..

[35] Charalambous H.P., Roussis P.C., Giannakopoulos A.E. (2017). Viscoelastic dynamic arterial response. Comp. Biol. Med.

[36] Sunagawa K., Kanai H., Koiwa Y., Nitta K., Tanaka M. (2001). Simultaneous measurement of vibrations on arterial wall upstream and downstream of arteriostenosis lesion and their analysis.. J. Med. Ultrason..

[37] Wang J.J., Liu S.H., Su H.M., Chang S., Tseng W.K. (2016). A vibration-based approach to quantifying the dynamic elastance of the superficial arterial wall.. Biomed. Eng. Online.

[38] Feng J., Khir A.W. (2010). Determination of wave speed and wave separation in the arteries using diameter and velocity.. J. Biomech..

[39] Wang J.J., Parker K.H. (2004). Wave propagation in a model of the arterial circulation.. J. Biomech..

[40] Khir A.W., O’Brien A., Gibbs J.S., Parker K.H. (2001). Determination of wave speed and wave separation in the arteries.. J. Biomech..

[41] Meinders J.M., Hoeks A.P. (2004). Simultaneous assessment of diameter and pressure waveforms in the carotid artery.. Ultrasound Med. Biol..

[42] Canić S., Hartley C.J., Rosenstrauch D., Tambaca J., Guidoboni G., Mikelić A. (2006). Blood flow in compliant arteries: an effective viscoelastic reduced model, numerics, and experimental validation.. Ann. Biomed. Eng..

[43] Womersley J.R. (1957). Oscillatory flow in arteries: the constrained elastic tube as a model of arterial flow and pulse transmission.. Phys. Med. Biol..

[44] Meinders J.M., Kornet L., Brands P.J., Hoeks A.P. (2001). Assessment of local pulse wave velocity in arteries using 2D distension waveforms.. Ultrason. Imaging.

[45] Cinthio M., Ahlgren A.R., Bergkvist J., Jansson T., Persson H.W., Lindström K. (2006). Longitudinal movements and resulting shear strain of the arterial wall.. Am. J. Physiol. Heart Circ. Physiol..

[46] Naghdi P.M., Cooper R.M. (1956). Propagation of elastic waves in cylindrical shells, including the effects of transverse shear and rotatory inertia.. J. Acoust. Soc. Am..

[47] Warriner R.K., Johnston K.W., Cobbold R.S. (2008). A viscoelastic model of arterial wall motion in pulsatile flow: Implications for Doppler ultrasound clutter assessment.. Physiological Measurement..

